# Functional heterogeneity in trophoblast stem cells derived from recurrent pregnancy loss products of conception

**DOI:** 10.1093/molehr/gaag033

**Published:** 2026-05-29

**Authors:** Qi Fu, Keisuke Kozai, Xingrao Ke, Joseph M Varberg, Sheng Xia, Ashley S Howard, Michael Lydic, Kristin Holoch, Courtney Marsh, Elin Grundberg, Kaela M Varberg

**Affiliations:** Fetal Health Center, Children’s Mercy, Kansas City, MO, USA; Fetal Health Center, Children’s Mercy, Kansas City, MO, USA; Fetal Health Center, Children’s Mercy, Kansas City, MO, USA; Biostatistics and Computational Biology Core, Division of Health Services and Outcomes Research, Children’s Mercy, Kansas City, MO, USA; Department of Research and Sponsored Projects Administration, Children’s Mercy, Kansas City, MO, USA; Fetal Health Center, Children’s Mercy, Kansas City, MO, USA; Department of Cell Biology and Physiology, University of Kansas Medical Center, Kansas City, KS, USA; Department of Obstetrics and Gynecology, University of Kansas Medical Center, Kansas City, KS, USA; Department of Obstetrics and Gynecology, University of Kansas Medical Center, Kansas City, KS, USA; Department of Obstetrics and Gynecology, University of Kansas Medical Center, Kansas City, KS, USA; Institute for Reproductive and Developmental Sciences, University of Kansas Medical Center, Kansas City, KS, USA; Department of Pediatrics, Genomic Medicine Center, Children’s Mercy, Kansas City, MO, USA; Fetal Health Center, Children’s Mercy, Kansas City, MO, USA; Department of Cell Biology and Physiology, University of Kansas Medical Center, Kansas City, KS, USA; Department of Obstetrics and Gynecology, University of Kansas Medical Center, Kansas City, KS, USA; Institute for Reproductive and Developmental Sciences, University of Kansas Medical Center, Kansas City, KS, USA

**Keywords:** recurrent pregnancy loss (RPL), trophoblast stem cells, products of conception (POC), placental development, trophoblast differentiation, cellular senescence

## Abstract

Recurrent pregnancy loss (RPL), defined as two or more consecutive miscarriages, affects 1–5% of couples attempting to conceive and most frequently occurs between 6 and 8 weeks of gestation, coinciding with critical stages of placental development. Despite this temporal association, placental contributions to RPL pathogenesis remain poorly understood. We hypothesized that trophoblast lineage development, including extravillous trophoblast (EVT) and syncytiotrophoblast (STB) differentiation, are disrupted in RPL. To test this hypothesis, we established and functionally characterized human trophoblast stem (TS) cell lines derived from products of conception (POC) obtained from consenting idiopathic RPL cases. Four TS cell lines were successfully derived from five POC samples, representing both normal and abnormal karyotypes. All established TS cell lines exhibited canonical stem-state trophoblast features, including epithelial morphology, ELF5 promoter hypomethylation, expression of trophoblast-specific microRNAs, and trophoblast marker expression. Despite this preserved identity, TS cell lines displayed marked heterogeneity in proliferation and differentiation capacity. TS cells derived from POC with abnormal karyotypes exhibited prolonged doubling times and impaired differentiation, while those from normal karyotypes more closely resembled cytotrophoblast-derived control TS cells. Although all TS cells formed morphologically comparable STB spheroids, functional deficits in human chorionic gonadotropin β secretion were observed in a cell-line-specific manner. EVT differentiation capacity varied substantially across POC-derived TS cell lines. Transcriptomic analyses revealed karyotype-associated transcriptional heterogeneity, including enrichment of senescence- and cell cycle-associated pathways across subsets of POC-derived TS cell lines. Together, these findings demonstrate that patient-specific TS cell lines retain trophoblast identity yet exhibit meaningful functional heterogeneity that becomes evident upon differentiation, underscoring the importance of functional validation in newly established *in vitro* placental cell lines. Overall, POC-derived TS cells are a powerful platform for investigating molecular mechanisms underlying RPL.

## Graphical Abstract

**Graphical Abstract:**
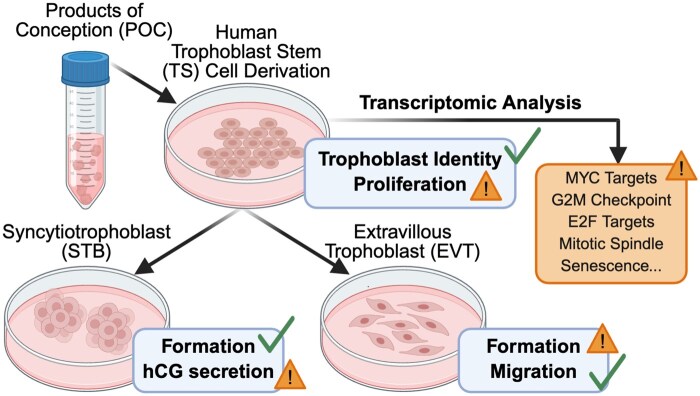
**Overview of trophoblast stem cell derivation and functional heterogeneity following recurrent pregnancy loss.** Schematic representation of human trophoblast stem (TS) cells derived from products of conception (POC) following recurrent pregnancy loss (RPL), highlighting trophoblast identity, proliferation dynamics, differentiation into syncytiotrophoblast (STB) and extravillous trophoblast (EVT) lineages, and associated transcriptomic alterations. TS, trophoblast stem; POC, products of conception; RPL, recurrent pregnancy loss; STB, syncytiotrophoblast; EVT, extravillous trophoblast; hCG, human chorionic gonadotropin; G2M, G2/M cell cycle transition; E2F, E2F transcription factors. Created in BioRender. Varberg, K. (2026) https://BioRender.com/ngxhvdm.

## Introduction

Recurrent pregnancy loss (RPL) remains a complex and poorly understood condition, particularly in idiopathic cases where no clear etiology is identified. A significant proportion of losses occur between 6 and 8 weeks of gestation: a critical window during which placental development is initiated (Brosens *et al.*, 2019). Defective placentation is a well-established contributor to early pregnancy loss (Rossant and Cross, 2001; Perez-Garcia *et al.*, 2018), yet the extent to which placental dysfunction underlies idiopathic RPL is still largely unexplored. The placenta may harbor key molecular and cellular signatures that could illuminate mechanisms of trophoblast lineage establishment and failure in RPL, but current diagnostic and research limitations hinder our ability to access and interpret this information. Despite known risk factors, such as maternal age, endocrine and uterine abnormalities, thrombophilia, infections, autoimmune conditions, and chromosomal defects (Turesheva *et al*., 2023), nearly half of RPL cases remain unexplained (Li *et al*., 2002; Practice Committee of the American Society for Reproductive Medicine, 2026; The ESHRE Guideline Group on RPL, 2022). Barriers including limited early pregnancy awareness, restricted access to care, and financial constraints further contribute to underdiagnosis. Advancing our understanding of placental contributions to RPL is essential for uncovering its pathogenesis and developing targeted therapeutic strategies.

Recent advances in placental *in vitro* modeling have opened new avenues for investigating the molecular mechanisms underlying early placental development. Notably, patient-specific human trophoblast stem (TS) cells can now be derived directly from placental tissue without the need for reprogramming. TS cells are powerful because they can be maintained in a stem-cell proliferative state or differentiated toward either syncytiotrophoblast (STB) or extravillous trophoblast (EVT) cell lineages. TS cells have been successfully established from first-trimester tissues, including cytotrophoblasts from terminations, chorionic villus sampling biopsies, and products of conception (POC) following pregnancy loss (Okae *et al.*, 2018; Saha *et al.*, 2020; Varberg *et al.*, 2025). Thus, TS cell culture enables the study of early placental events that were previously inaccessible. However, it remains unclear how the derivation efficiency and TS cell phenotype may vary across different pregnancy pathologies, and whether these cells retain disease-specific characteristics post-derivation is still under active investigation.

In this study, we established and characterized four patient-specific human TS cell lines derived from POC following idiopathic RPL. Although all established TS cell lines exhibited hallmark stem-state features comparable to cytotrophoblast-derived controls, functional heterogeneity was evident. Proliferation rates varied by karyotype, with TS cells derived from karyotypically normal POCs resembling controls and those from karyotypically abnormal POCs exhibiting slower growth. Consistent with this heterogeneity, RPL-derived TS cell lines showed cell line-specific deficits in differentiation toward both EVT and STB lineages. We hypothesize that impaired TS cell function may contribute to pregnancy failure in idiopathic RPL, though further studies are needed to elucidate mechanisms and establish causality. Our findings emphasize the importance of validating TS cells beyond morphology, incorporating functional assessments. This work expands the collection of well-characterized human TS cells available for investigating placental development in failed pregnancies. While the limited number of cell lines is a constraint, ongoing efforts aim to broaden this resource. We propose that integrating data across diverse patient-specific cell lines will offer new insights into placental mechanisms in both healthy and pathological contexts.

## Materials and methods

### Study approval

POC tissues were obtained from consenting idiopathic RPL patients through the Children’s Mercy Genomic Answers for Kids Protocol (IRB No. 11120514). All work relating to human subjects was reviewed and approved by the Children’s Mercy Institutional Review Board.

### Tissue collections and inclusion criteria

POC tissues were collected between 6 and 8 weeks of gestation following miscarriage in patients experiencing RPL. All study participants had transvaginal ultrasound to confirm diagnosis of miscarriage and had opted for dilation and curettage procedure as a desired treatment option for surgical evacuation of miscarriage. Informed written consent was obtained from all study participants. Procedures were performed by reproductive endocrinologists in the Department of Obstetrics and Gynecology, University of Kansas Medical Center. POC tissue from clinically unexplained (except chromosomal abnormality) RPL cases were included in this study (Table 1). We included cis-women 18 years or older who had one or more miscarriages in the study. Participants were excluded from the study if pregnancy was conceived with use of donor gametes or embryo(s). Cis women were also excluded from participating if they had any of the following conditions: uterine anomalies, coagulopathy, balanced translocation, autoimmune conditions, morbid obesity, tobacco use within 3 months of conception, history of gonadotoxic therapy, endometrial hyperplasia or cancerous condition of the female reproductive tract, submucosal fibroid(s), pathology confirmed or suspected endometriosis, pathology confirmed or suspected adenomyosis, endometrial polyp(s), pathology confirmed as acute or chronic endometritis, hydrosalpinx communicating with endometrial cavity, intrauterine adhesions, poorly controlled endocrinopathy (diabetes mellitus with HgbA1c >6.5%, hypothyroidism with TSH ≥4.5 mU/l), active rheumatologic disease, and/or HIV.

**Table 1.** Cell line-associated clinical information.

TS cell line	POC cytogenetics	TS cell cytogenetics	TS model derivation outcome	Sex	Gestational age	Prior miscarriages(#)	Maternal/paternal age	Additional information
R001	arr(22)x3	N/A	Failed*	M	6w1d	1	39/42	Intrauterine Insemination
R002	arr(22)x3[0.6]	arr(22)x3[0.5]	Success	M	6w1d	4	41/39	N/A
R003	arr(15)x3	arr(15)x3	Success	F	8w0d	1	41/32	Letrozole
R004	arr(X , 1-22)x2	arr(X , 1-22)x2	Success	F	7w0d	3	28/28	N/A
R005	arr(X , 1-22)x2	arr(X , 1-22)x2	Success	F	6w2d	1	33/28	N/A

arr, array technique used; d, day; F, female; M, male; POC, product of conception; TS, trophoblast stem; w, week.

* A TS cell line was not established from POC tissue. Cells did not transition into phase two of the derivation process marked by exponential cell proliferation (Supplementary Fig. S3).

### Derivation and culture of human TS cells

POC tissue was enzymatically digested in 1.25 U/ml of Dispase II (D4693-1G, Sigma-Aldrich, St. Louis, MO, USA), 0.4 mg/ml of Collagenase IV (C5138-100mg, Sigma-Aldrich), and 80 U/ml of DNase I (DN25-1G, Sigma-Aldrich) in Hank’s Balanced Salt Solution (14170112, Thermo Fisher Scientific, Waltham, MA, USA) for 15∼30 min at 37 °C with agitation. Single cell suspensions were prepared and viable cell aliquots cryopreserved in the Children’s Mercy Genomic Medicine Center. Cryopreserved, viable single cell suspensions were thawed and cultured following published protocols for human TS cell culture (Okae *et al.*, 2018; Varberg *et al.*, 2021, 2023). Viable cells were plated in 24- (353504, Corning, Glendale, AZ, USA) or 6-well tissue culture-treated dishes (140685, Thermo Fisher Scientific), coated with 5 μg/ml human collagen type IV (C5533, Sigma-Aldrich) diluted in phosphate-buffered saline (PBS). The choice of plate format was based on the number of live cryopreserved cells. Cells were cultured in 500 μl (24-well) or 2 ml (6-well) of complete TS Cell Medium [Dulbecco’s Modified Eagle Medium/Ham’s F-12 (DMEM/F12; 11320033, Thermo Fisher Scientific), 50 U/ml penicillin, 50 μg/ml streptomycin, 0.3% bovine serum albumin (BSA; BP9704100, Thermo Fisher Scientific), 1% Insulin-Transferrin-Selenium-Ethanolamine (ITS-X) solution (vol/vol; 51500056, Thermo Fisher Scientific)], 0.2% Fetal Bovine Serum (FBS; 16141002, Thermo Fisher Scientific), 0.1 mM 2-mercaptoethanol (444203, Sigma-Aldrich), 1.5 μg/ml L-ascorbic acid (A8960, Sigma-Aldrich), 50 ng/ml Epidermal Growth Factor (EGF; E9644, Sigma-Aldrich), 2 μM CHIR99021 (04-0004, Reprocell, Beltsville, MD, USA), 0.5 μM A83-01 (04-0014, Reprocell), 1 μM SB431542 (04-0010, Reprocell), 0.8 mM valproic acid (VPA) (P4543, Sigma-Aldrich), and 5 μM Y27632 (04-0010-10, Reprocell)] (Okae *et al.*, 2018). Cells attached to the culture surface within 1 day. Culture medium was replaced with fresh complete TS cell culture medium after initial attachment and every 2 days thereafter. Colonies emerged within 2–5 days. Time to first passage was unique to each sample and determined by the colony density and confluency of fibroblasts surrounding the colonies. On average, the first passage occurred 11 days post-plating. To passage, cells were washed with PBS and detached with TrypLE Express Enzyme (12604021, Thermo Fisher Scientific). Cells were replated in complete TS cell culture medium in collagen-coated 24- or 6-well plate formats. Following line derivation and for routine culture, TS cells were cultured in dishes pre-coated with 5 μg/ml human collagen type IV. TS cells were maintained in a complete TS cell culture medium which was replaced every 2 days of culture. Cytotrophoblast-derived TS cell lines CT27 (XX) and CT29 (XY) were used as reference controls (Okae *et al.*, 2018).

### Population doubling time

Population doubling times were calculated as [culture time × (ln2)]/[ln (initial cell number/final cell number)] and were measured between passage (P)15 and P20. Cumulative cell number represents the cumulative number of cells started from the initial culture until the cell line derivation around passages 8–11.

### Cytogenetic analysis

Cytogenetic analysis was performed on both the POCs and the newly derived TS cell lines using the CytoScan HD microarray platform (Thermo Fisher Scientific), and chromosomal abnormalities were described according to the 2024 edition of the International System for Human Cytogenomic Nomenclature (Hastings *et al.*, 2024).

### miRNA isolation, cDNA preparation, and quantitative real-time PCR

Total RNA was isolated using AllPrep DNA/RNA Mini Kits (80204, Qiagen, Germantown, MD, USA) following the manufacturer’s instructions with modifications to capture small RNAs simultaneously. One modification was to use 1.5× volume of 100% ethanol to the flowthrough instead of 1× volume of 70% ethanol in the first step of RNA isolation. cDNA synthesis was performed with TaqMan Advanced miRNA cDNA Synthesis kit (A28007, Thermo Fisher Scientific). RT-qPCR was performed using TaqMan™Fast Advanced Master Mix (4444556, Thermo Fisher Scientific) and targeted miRNAs hsa-miR-517a-3p, hsa-miR-517-5p, hsa-miR-526b-3p, and housekeeping miRNA hsa-miR-103a-3p (479485_mir, 478996_mir, and 478253_mir; TaqMan™ Advanced miRNA Assays, Thermo Fisher Scientific). Relative expression of each transcript was calculated using the ΔΔCT method and normalized to hsa-miR-103a-3p. TS cell lines CT27 and CT29 (Okae *et al.*, 2018) were used as reference lines. Induced pluripotent stem cells (iPSCs) were used as a negative control cell line.

### Bisulfite pyrosequencing

Genomic DNA was isolated using the AllPrep DNA/RNA Mini Kit (80204, Qiagen) following the manufacturer’s instructions, and DNA was bisulfite converted using the EpiTect Plus Bisulfite Kit (59124, Qiagen) according to the manufacturer’s instructions. Following bisulfite conversion, bisulfited-treated DNA equivalent to 20 ng of the DNA prior to bisulfite treatment was used per PCR. Primers for PCR and sequencing were designed by using PyroMark Assay Design 2.0 software (Qiagen). Three amplicons were studied to cover a total of 10 CpG sites (Table 2). PCR conditions were 95 °C for 10 min, followed by 50 cycles of 94 °C for 30 s, 62 °C for 30 s, and 72 °C for 30 s with magnesium 3 mM. To avoid PCR artifacts that generate bias on the methylation status of CpGs (Shen *et al.*, 2007), we optimized annealing temperature by using human methylated genomic DNA standards (EpigenDx, Inc., Hopkinton, MA, USA) as the DNA template. The annealing temperature used for the final experiments was that which generated a percentage of CpG methylation closest to the known percentage of methylation in the methylation standard DNA. Pyrosequencing was performed using Q48 Auroprep (Qiagen) in conjunction with PyroMark Q48 Advanced CpG Reagents (Qiagen) and PyroMark Q48 Magnetic Beads (Qiagen). The results were analyzed using Pyromark Q48 Autoprep software (Qiagen). TS cell lines CT27 and CT29 (Okae *et al.*, 2018) were used as reference lines. iPSCs were used as a cell type methylation positive control for these CpG sites.

**Table 2.** ELF5 promoter bisulfite pyrosequencing primers.

CpG sites covered	Primer sequence
CpG (−65, −45, −26, −6) relative to TSS	Forward: 5′-GGGGTGAGTTGAGTATAAAAGTAGGAT-3′ Reverse:/5′Biosg/AAAAAACAAACCCTCCCAACACCA-3′ Sequencing: 5′-TGAGTTGAGTATAAAAGTAGGATAA-3′
CpG (−130) relative to TSS	Forward: 5′-AGTTAGTTTGGGAGAGAGGTAG-3′ Reverse:/5′Biosg/CATATACAAAAAATTTACCTTTATCCTACT-3′ Sequencing: 5′-TTTTGGGTTGGGAGT-3′
CpG (−299, −292, −228, −224, −217) relative to TSS	Forward: 5′-AGTTTAGGGGATTTAGTAAATAAGTAGA-3′ Reverse:/5′Biosg/AAATCTTAACACTAAACCTATAATACTCT-3′ Sequencing: 5′-AGAAAATTTTTGTTTTTGGGA-3′

TSS, transcription start site; 5′Biosg, 5′ Biotin.

### Immunocytochemical analysis

Cells were fixed with 4% paraformaldehyde (15714, Electron Microscopy Sciences, Hatfield, PA, USA) for 15 min at room temperature. Fixed cells were incubated with primary antibody against HLA-G (1:400, ab52455, Abcam, Waltham, MA, USA), cytokeratin 7 (KRT7; 1:50, ab183344, Abcam), GATA binding protein 3 (GATA3; 1:25, AF2605, R&D Systems, Minneapolis, MN, USA), transcription factor AP-2 gamma (TFAP2C; 1:200, No. 2320, Cell Signaling Technology, Danvers, MA, USA), and vimentin (VIM; 1:400, No. 5741, Cell Signaling Technology), followed by fluorophore-conjugated secondary antibody of immunoglobulin of corresponding species. The cells were then mounted with 4′,6-diamidino-2-phenylindole (DAPI) mounting medium (H-1200-10, Vector Laboratories, Newark, CA, USA) to co-stain nuclei. Fluorescence images were captured on a Nikon W1 spinning disk confocal microscope (Nikon, Tokyo, Japan).

### Flow cytometry

Cells were dissociated with TrypLE, passed through a 70 μm mesh filter, and suspended in Flow Cytometry Staining Buffer (00-4222-57, Thermo Fisher Scientific). For flow cytometric analysis of HLA-ABC, cells were incubated with an FITC-conjugated anti-HLA-ABC monoclonal antibody (1:20, W6/32, 11-9983-42, Thermo Fisher Scientific) for 30 min at room temperature. FITC-conjugated mouse IgG2a Κ (11-9983-42, Thermo Fisher Scientific) was used as isotype control. Flow cytometry was carried out using an Attune NxT Acoustic Focusing Cytometer (Thermo Fisher Scientific), and the data were analyzed using FlowJo software (v10.8.1; BD Biosciences, Ashland, OR, USA).

### STB differentiation

To induce STB cell differentiation, we plated TS cells into 6 cm petri dishes (not tissue culture-treated, 351007, Corning) at a density of 200 000 cells per dish and cultured in three-dimensional STB (ST3D) medium [DMEM/F12 (11320033, Thermo Fisher Scientific), 50 U/ml penicillin, 50 μg/ml streptomycin, 0.1 mM 2-mercaptoethanol (444203, Sigma-Aldrich), 0.3% BSA (BP9704100, Thermo Fisher Scientific), 1% ITS-X solution (vol/vol; 51500056, Thermo Fisher Scientific)], 4% Knockout Serum Replacement (KSR; 10828028, Thermo Fisher Scientific), 2.5 μM Y27632 (04-0012, Reprocell), 2 μM forskolin (F6886, Sigma-Aldrich), and 50 ng/ml EGF (E9644, Sigma-Aldrich)] (Okae *et al.*, 2018). On Day 3 of cell differentiation, 3 ml of fresh ST3D medium was added to the culture dishes. Cells were analyzed on Day 6 of STB cell differentiation. TS cell lines CT27 and CT29 (Okae *et al.*, 2018) were used as reference lines.

### STB spheroid surface and volume quantification

STB cell spheroids were fixed with 4% paraformaldehyde for 20 min, permeabilized with 0.3% Triton X-100 (A16046, Alfa Aesar, Haverhill, MA, USA) in PBS for 10 min, blocked with 2% FBS/PBS for 1 h at room temperature, and incubated with 1:100 diluted PE-conjugated anti-SDC1 (130-119-840, Biotec, Auburn, CA, USA) and 1:10 diluted rabbit anti-chorionic gonadotropin (GA50861-2, Dako Omnis, Agilent, Santa Clara, CA, USA) overnight at 4 °C. Spheroids were incubated with Alexa Fluor 488 conjugated goat anti-rabbit IgG for 1 h at room temperature. The spheroids were transferred onto glass microscope slides and cover slipped with DAPI mounting medium (H-1200-10, Vector Laboratories). For imaging, Imaris software (v10.2.0; Bitplane, Zurich, Switzerland) 3D reconstruction and parametric analysis Z-stack images were acquired using a Nikon W1 spinning disk confocal microscope at 10× magnification with 3×3 fields. Each sample was imaged from three to five spots randomly and processed with Imaris (Bitplane) for 3D reconstruction. The spheroid count, surface, and volume were calculated based on intensity of all channels on Z-stacks composed of individual images (Supplementary Fig. S1). The analysis involved examining at least 150 spheroids per sample, derived from three independent experiments. Spheroids incubated with secondary antibody only were used as a negative control.

### Total RNA isolation and RT-qPCR

Total RNA was isolated using RNeasy Plus mini kit (74136, Qiagen). cDNA was synthesized using the High-Capacity cDNA Reverse Transcription Kit (4368813, Thermo Fisher Scientific) and diluted 10× with ultra-pure distilled water. qPCR was performed using PowerSYBR Green PCR Master Mix (4367659, Thermo Fisher Scientific) and primers (250 nM each). RT-qPCR primer sequences are presented in Table 3. Amplification and fluorescence detection were measured with a QuantStudio 12K Flex Real-Time PCR System (Thermo Fisher Scientific). An initial step (95 °C, 10 min) preceded 40 cycles of a two-step PCR (92 °C, 15 s; 60 °C, 1 min) and was followed by a dissociation step (95 °C, 15 s; 60 °C, 15 s; 95 °C 15 s). The ΔΔCT method was used for relative quantification of the amount of mRNA for each sample normalized to the housekeeping gene TATA-box binding protein (TBP).

**Table 3.** RT-qPCR primers (human).

Gene	Forward primer	Reverse primer
*TBP*	5′-CCCATGACTCCCATGACC-3′	5′-TTTACAACCAAGATTCACTGTGG-3′
*TEAD4*	5′-CAGGTGGTGGAGAAAGTTGAGA-3′	5′-GTGCTTGAGCTTGTGGATGAAG-3′
*LRP2*	5′-CTGCTCCTGGCTCTCGTC-3′	5′-TCCCATCACACCTCCAGTCT-3′
*LIN28A*	5′-CCCCCAGTGGATGTCTTTGT-3′	5′-ATGGATTCCAGACCCTTGGC-3′
*CGB7*	5′-CAAACCCGAGGAATAAAGCCAG-3′	5′-ATGCTTCGGCCACGGT-3′
*SDC1*	5′-CTATTCCCACGTCTCCAGAACC-3′	5′-GGACTACAGCCTCTCCCTCCTT-3′
*CYP11A1*	5′-AGACGGGCACACACAAAGTC-3′	5′-CATAAACCGACTCCACGTTGC-3′
*HLA-G*	5′-CCACCACCCTGTCTTTGACTAT-3′	5′-ACGTCCTGGGTCTGGTCCT-3′
*MMP2*	5′-TGGCACCCATTTACACCTACAC-3′	5′-ATGTCAGGAGAGGCCCCATAGA-3′
*CCR1*	5′-AGTACCTGCGGCAGTTGTTC-3′	5′-AAGGGGAGCCATTTAACCAG-3′

### RNA sequencing

Total RNA was isolated from cultured stem state TS cells using RNeasy Plus mini kit (74136, Qiagen). Passage (P) numbers for cultured TS cells were as follows: CT27 (P22), CT29 (P18), R002–R005 (P12). Stranded total RNA-sequencing was performed on the Illumina NovaSeq X Plus Sequencing System in the Genomic Medicine Center at Children’s Mercy. Quality control was completed with the RNA Screen Tape Assay kit (5067-5576, Agilent Technologies) on the Agilent TapeStation 4200. Total RNA (500 ng) was processed using the Illumina TruSeq Stranded Total RNA Library Prep Gold (20020598, Illumina) in the following steps: (i) ribosomal RNA (rRNA) depletion using biotinylated, target-specific oligos combined with Ribo-Zero rRNA, (ii) fragmentation, (iii) reverse transcription/first strand synthesis, (iv) second strand synthesis, (v) A-tailing, (vi) Unique Dual Index (UDI) adaptor ligation, and (vii) library amplification. Library validation was performed with the D1000 Screen Tape Assay kit (5067-5582, Agilent) on the Agilent Tape Station 4200. Library concentrations were determined with a Qubit 4 Fluorometer (Thermo Fisher Scientific). Libraries were pooled based on equal molar amounts and the multiplexed pool was quantified using a Qubit 4 Fluorometer and sized using the D1000 Screen Tape Assay kit. The RNA-Seq library pool was adjusted to 3 nM for multiplexed sequencing. Pooled libraries were denatured with 0.2 N NaOH (0.04 N final concentration) and diluted to 90 pM. Onboard clonal clustering of the patterned flow cell was performed using the NovaSeq X Plus 25B (300 cycle, 20104706, Illumina). A 2×151 cycle sequencing profile with dual index reads was completed using the following sequence profile: Read 1–151 cycles×Index Read 1–17 cycles×Index Read 2–8 cycles×Read 2–151 cycles.

### Data processing and analyses

Sequencing files were processed using the nf-core/rnaseq pipeline (v. 3.19.0; doi: 10.5281/zenodo.1400710) from the nf-core collection of workflows (Ewels *et al.*, 2020) using Nextflow v25.04.6 (Di Tommaso *et al.*, 2017), with all default parameter values. Briefly, adapter sequences were trimmed using Trim Galore! (v. 0.6.10) with Cutadapt (v. 4.9). Trimmed reads were mapped to the GRCh38 (release 114) human reference genome with STAR (v. 2.7.10a) and quantified using RSEM (v. 1.3.1). The RSEM gene-level counts matrix was used for downstream analysis using DESeq2 (v. 1.46.0) in R (v. 4.4.0) using targets (v. 1.11.3) (Love *et al.*, 2014). Differentially expressed (adjusted *P*-value <= 0.05, no log2-fold change threshold) genes were identified using DESeq with a design model incorporating sex as a covariate. Results for each contrast of interest were extracted with log-fold change shrinking using the ‘ashr’ model (Stephens, 2017). The DEseq2 metadata are provided in Supplementary Table S1, along with the normalized counts matrix (normalized using the variance stabilizing transformation) in Supplementary Table S2 and full differential expression results in Supplementary Table S3.

Gene set enrichment testing was performed using limma’s camera test (limma::cameraPR()) using the log2-fold change value as the test statistic for 50 Hallmark gene sets supplemented with 23 senescence-related pathways present in the MSigDB human ‘C2’ gene set collection, accessed using msigdbr (v. 25.1.1) (Wu and Smyth, 2012).

### Human chorionic gonadotropin ELISA

Conditioned medium was collected following 6 days of STB culture. Human chorionic gonadotropin β (hCGβ) levels were measured using an ELISA kit (DY008B and DY9034-05, R&D systems/Bio-techne), following the manufacturer’s protocol.

### EVT cell differentiation

EVT cell differentiation was induced by plating TS cells onto 6-well plates pre-coated with 1 μg/ml of mouse type IV collagen at a density of 80 000 cells per well (Okae *et al.*, 2018). Cells were cultured in EVT Differentiation Medium [DMEM/F12 (11320033, Thermo Fisher Scientific), 0.1 mM 2-mercaptoethanol, 50 U/ml penicillin, 50 μg/ml streptomycin, 0.3% BSA (BP9704100, Thermo Fisher Scientific), 1% ITS-X solution (vol/vol; 51500056, Thermo Fisher Scientific)], 100 ng/ml neuregulin 1 (NRG1; 5218SC, Cell Signaling Technology), 7.5 μM A83-01 (04-0014, Reprocell), 2.5 μM Y27632 (04-0012, Reprocell), 4% KSR (10828028, Thermo Fisher Scientific), and 2% Matrigel (CB-40234, Thermo Fisher Scientific). On Day 3 of EVT cell differentiation, the medium was replaced with EVT Differentiation Medium excluding NRG1 and with a reduced Matrigel concentration of 0.5%. On Day 6 of EVT cell differentiation, the medium was replaced with EVT Differentiation Medium with a Matrigel concentration of 0.5% and excluding NRG1 and KSR. Cells were analyzed on Day 8 of EVT cell differentiation. TS cell lines CT27 and CT29 (Okae *et al.*, 2018) were used as reference lines.

### Time-lapse live imaging and data analysis

For time-lapse live imaging, EVT cells were dissociated, stained with 0.5× SPY505-DNA nuclear stain (30874, Cytoskeleton, Inc., Denver, CO, USA), and re-seeded in triplicate into a 48-well tissue culture-treated plate on Day 6 of EVT differentiation. Imaging was initiated immediately after seeding using a Nikon W1 spinning disk confocal microscope (Nikon, Japan), capturing both green fluorescent protein channel and differential interference contrast images over a 44-h period. The acquired imaging data were converted using Imaris software (Bitplane). A machine learning model was trained within the Imaris software (Bitplane) to identify and classify elongated versus non-elongated EVT cells (Supplementary Fig. S2). To track cell migration, we masked EVT cells using a green fluorescent protein nuclear signal and used the nuclei as reference points for tracking cell movement. The percentage of EVT cells relative to the total cell number was calculated at the end of imaging (Day 8 of EVT differentiation).

### Protein isolation and immunoblotting

Total cell lysates were prepared in radioimmunoprecipitation assay buffer (89900, Thermo Fisher Scientific) and quantified using a Pierce BCA Protein Assay Kit (23227, Thermo Fisher Scientific). HLA-G protein quantification was completed using capillary immunoassay on the Jess Simple Western system (ProteinSimple, San Jose, CA, USA). 1:1000 dilution of anti-HLA-G (ab52455, Abcam) was used. Total protein normalization was used as protein loading control.

### Stromal cell derivation

Stromal cells were derived from POC tissue R001. Stromal cells that remained following TS cell line derivation failure were cultured in stromal cell medium [DMEM/F12 (11320033, Thermo Fisher Scientific), 50 U/ml penicillin, 50 μg/ml streptomycin, and 10% FBS (A5256801, Thermo Fisher Scientific)]. The medium was replaced every 3 days. A stromal cell line was established by continued passaging for five to six passages, using a 1:6 split ratio at each passage.

### Statistical analyses

All analyses were conducted using GraphPad Prism (v10.3.0; GraphPad Software, Boston, MA, USA). Data were first assessed for normality or lognormality using the Shapiro–Wilk test and the Kolmogorov–Smirnov test with the Dallal–Wilkinson–Lillie correction for the *P*-value. All datasets, except for EVT migration distance, followed a normal distribution. Logarithmic EVT migration distance was analyzed using the Kruskal–Wallis test followed by Dunn’s multiple comparisons test for *P*-value adjustment. One-way ANOVA was used to analyze cell population doubling time, miRNA expression, CpG methylation, STB spheroid surface area and volume, hCGβ ELISA, and HLA-G western-blot data. mRNA expression data comparing two cell types were analyzed using two-way ANOVA with Tukey’s *post hoc* test to evaluate main and interaction effects, with cell line and cell type as independent variables. *P *< 0.05 was considered statistically significant.

## Results

### Patient-specific TS cells were derived from POC tissue following RPL

Overall, four new TS cell lines were established from five unique POC tissue samples, representing an 80% success rate. The high rate of success supports the feasibility of using POC as a source for TS cell derivation. The cause of failed derivation could be attributed to several different factors, including cell composition of the POC tissue, the timing of the failed pregnancy, or an inherent defect in the TS cells. Additional studies and increased sample numbers are needed to understand why certain samples fail to produce TS cell lines.

Each POC tissue sample was associated with clinical and cytogenetic data (Table 1). POC tissues were obtained between gestational ages of 6 weeks, 1 day and 8 weeks, 0 days. Corresponding fetal sex from the pregnancy losses included two male fetuses (R001 and R002) and three female fetuses (R003, R004, and R005). The maternal age from the cases ranged from 28 to 41 years, while paternal ages ranged from 28 to 42 years. Some patients had experienced one prior clinically documented miscarriage (R001, R003, and R005), while others had experienced up to three (R004) or four (R002) prior miscarriages. Clinical cytogenetic reports indicated that three cases had a reported pathogenic finding (R001, R002, and R003). Tissues from R001 and R002 showed complete or partial chromosome 22 trisomy, respectively. Trisomy of chromosome 15 was found in the case of R003. Tissues from R004 and R005 had normal karyotypes. Thus, 60% of cases had a chromosomal abnormality, while 40% had normal karyotypes. POC tissues encompass a wide range of clinical backgrounds, offering new opportunities to uncover mechanisms underlying the pathogenesis of RPL.

**Figure 1.**
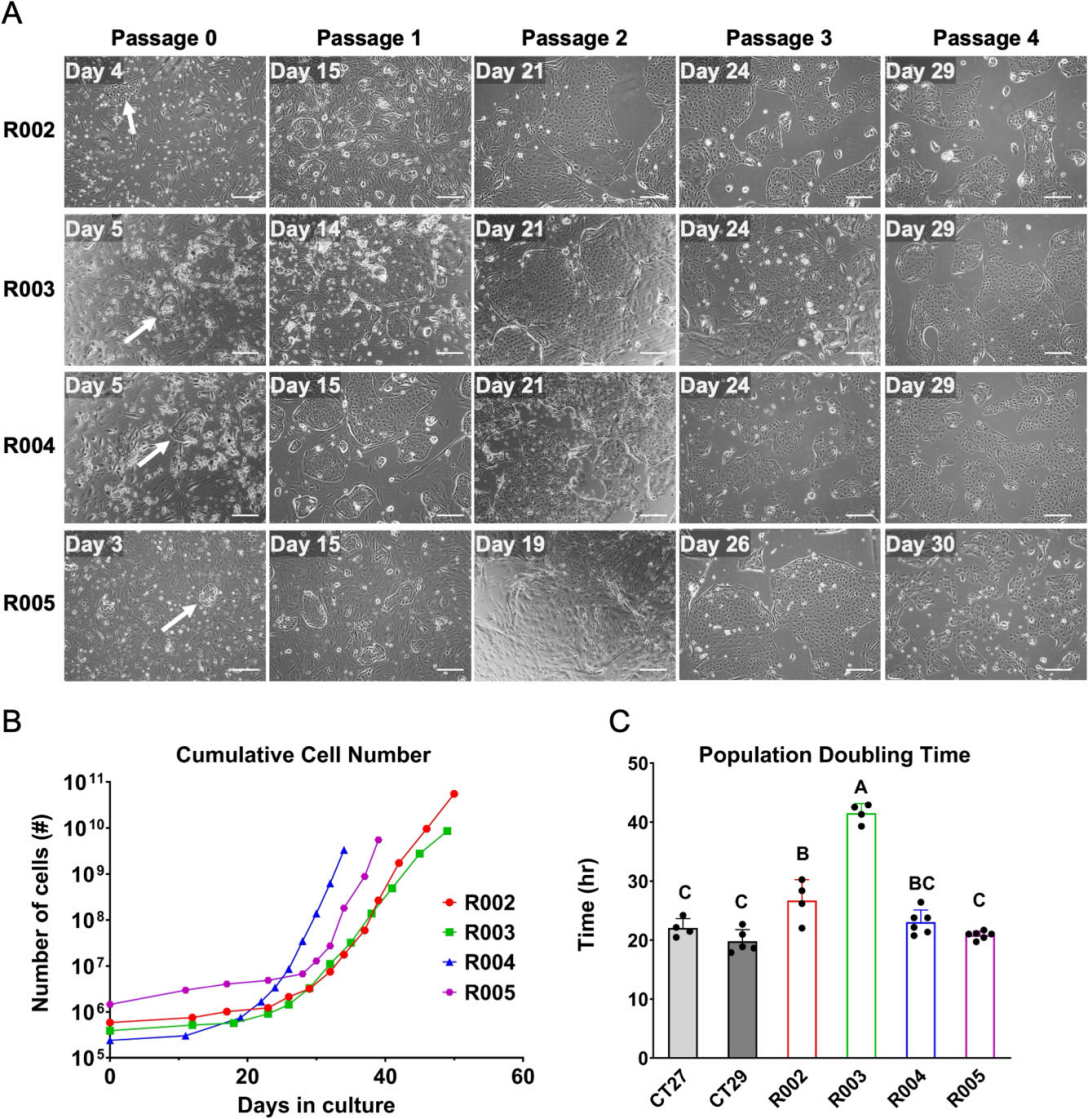
**Growth kinetics during trophoblast stem cell derivation from products of conception.** Products of conception (POC)-derived trophoblast stem (TS) cells exhibit variable proliferation and growth dynamics during derivation. (**A**) Representative phase contrast images of TS cell lines derived from POC samples (R002–R005) at defined stages of derivation, including pre-passage (P0) and passages 1–4. Days in culture are indicated in each panel. White arrows denote colony emergence. Scale bar, 250 μm. (**B**) Cumulative cell number from initial plating through completion of TS cell line derivation. (**C**) Population doubling time of cytotrophoblast-derived TS cells (CT27, CT29) and POC-derived TS cells (R002–R005), measured between passages 15–20. Values represent mean ± standard deviation (SD), n = 4–6 independent experiments. Groups without a shared letter are significantly different (one-way ANOVA, *P *< 0.05). POC, products of conception; TS, trophoblast stem; P, passage.

Viable, dissociated single cells from POC tissue were plated in TS cell culture conditions (Okae *et al.*, 2018). Cells attached within the first day of culture, and colonies emerged prior to initial passaging, typically 3–5 days after plating (Fig. 1A). Time to first passage was dependent on cell density and colony expansion but often occurred between 12- and 15-days post-plating. Cell heterogeneity decreased with each passage due to naturally occurring, media-induced growth selection. Independent epithelial TS cell colonies were evident after passage three, or ∼24 days in culture (Fig. 1A). Thus, the first 3–4 weeks in culture, or the first phase of TS cell derivation, primarily involved colony emergence resulting in a consistent but modest increase in total cell number (Fig. 1B).

The second phase of TS cell derivation was evident by the sudden initiation of exponential cell growth between 3 and 4 weeks in culture (Fig. 1B). Growth trajectories, based on cumulative cell numbers, varied between TS cell lines. Line R004 showed the most rapid expansion rate, despite the lowest number of cells at initial plating. Lines R002, R003, and R005 exhibited similar growth rates from 30 to 40 days in culture. After 40 days, growth rates started to diverge. Line R001 grew at a similar pace to the other lines until passage two, after which colony expansion halted, and cell numbers rapidly decreased (Supplementary Fig. S3A and B). Of note, the timing of R001 cell line derivation failure occurred at the transition point between phase one and two, just after 20 days in culture (Supplementary Fig. S3B). The ability of TS cells to undergo exponential cell growth represented a critical step in cell line derivation success. Overall, growth trajectory assessment during TS cell line derivation revealed a consistent bi-phasic pattern. Cell expansion rates were variable across individual samples and seemingly independent of starting cell number.

To quantify cell proliferation following TS cell line derivation, we calculated cell population doubling times between passages 15 and 20. Consistent with the growth trajectories observed during the derivation phase, population doubling times varied across cell lines (Fig. 1C). Compared to cytotrophoblast-derived TS cells, CT27 and CT29, at equivalent passage number, RPL POC-derived TS cell lines displayed varying rates of proliferation. CT27 and CT29 TS cells had population doubling times of 22.1 and 19.8 h, respectively. Line R005 proliferated at the same rate with population doubling every 20.85 h. R004 and R002 were slightly slower, with rates of 23.1 and 26.7 h, respectively. R003 proliferation was significantly slower than the other cell lines, with a doubling time of 41.5 h (*P* < 0.05; Fig. 1C). Phenotypic variability was maintained across patient-specific TS cell lines following line establishment and 20 passages in culture (data not shown).

### POC-derived TS cells exhibited defining features of trophoblast identity

**Figure 2.**
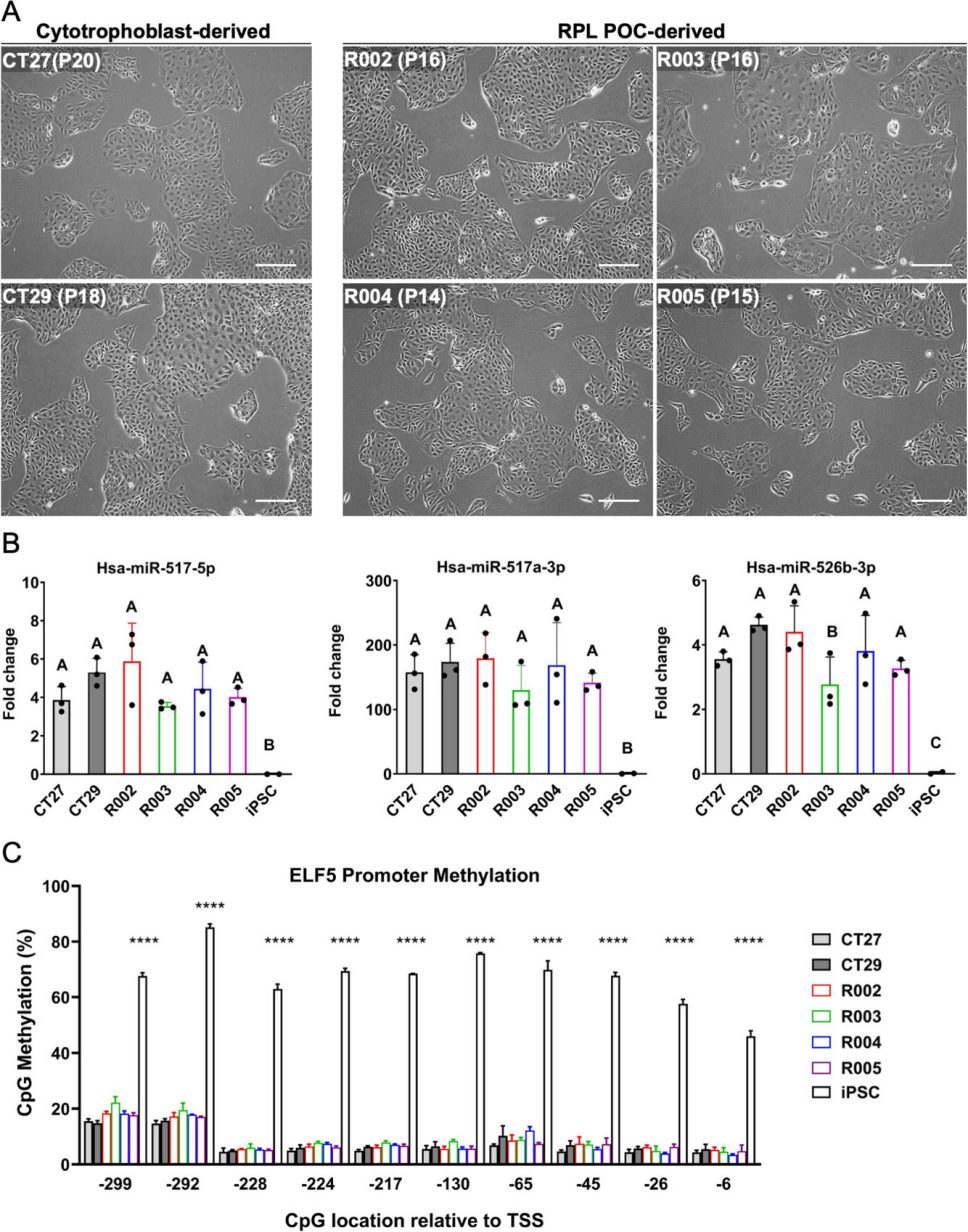
**Trophoblast identity is preserved in trophoblast stem cells derived from products of conception.** Products of conception (POC)-derived trophoblast stem (TS) cells retain canonical features of trophoblast identity. (**A**) Representative phase contrast images of cytotrophoblast-derived (CT27, CT29) and POC-derived TS cells (R002-R005) maintained in stem state culture (passages 14–20). Scale bar, 250 μm. (**B**) Expression of chromosome 19 microRNA cluster (C19MC) components (has-miR-517-5p, hsa-miR-517a-3p, and hsa-miR-526b-3p) relative to induced pluripotent stem cells (iPSCs). Expression is normalized to hsa-miR-103a-3p. Values represent mean ± standard deviation (SD), n = 3 independent experiments. Groups without a shared letter differ significantly (one-way ANOVA, *P *< 0.05). (**C**) DNA methylation levels at 10 CpG within the ELF5 promoter. Values represent mean ± SD, n = 3. Comparisons between TS cells and iPSCs were performed at matched CpG sites (*P *< 0.05). POC, products of conception; TS, trophoblast stem; iPSC, induced pluripotent stem cell; miR, microRNA; ELF5, E74-like ETS transcription factor 5.

Starting at the earliest stages of TS cell propagation, including the initial emergence of the first colonies, TS cells grew in discrete highly proliferative colonies. Colony growth patterns were maintained beyond the derivation phase and into the exponential growth and line maintenance phases. All POC-derived TS cells displayed epithelial cell cobblestone morphology and colony growth patterns (Figs 1A and 2A). Cell morphology and colony growth patterns were consistent with those of cytotrophoblast-derived TS cells (CT27/CT29) at equivalent passage numbers.

The C19MC miRNA cluster was consistently expressed across different TS cells, with minimal expression in other cell types, such as iPSCs (Fig. 2B). Therefore, expression of the C19MC miRNA is regarded as one of the widely accepted defining criteria for human TS cells (Lee *et al.*, 2016). Expression levels of Hsa-miR-517-5p, Hsa-miR-517a-3p, and Hsa-miR-526b-3p were compatible between POC-derived (R002-R005) and cytotrophoblast-derived TS cells (CT27/CT29). Hsa-miR-517a-3p exhibited the highest fold change increase when normalized to control miRNA hsa-miR-103a-3p. R003 TS cells expressed significantly less Hsa-miR-526b-3p compared with other TS cells (*P *< 0.05; Fig. 2B).

Another feature of trophoblast cells is hypomethylation of the E74-Like ETS Transcription Factor 5 (ELF5) promoter (Lee *et al.*, 2016). CpG methylation was assessed at 10 distinct sites (−6 to −299 bp) upstream of the transcription start site of the proximal ELF5 promoter (Fig. 2C). While the ELF5 promoter is methylated in iPSCs, these same CpG sites were hypomethylated in TS cells (*P *< 0.05; Fig. 2C). No differences in the percent methylation were observed at any CpG site tested across TS cells.

**Figure 3.**
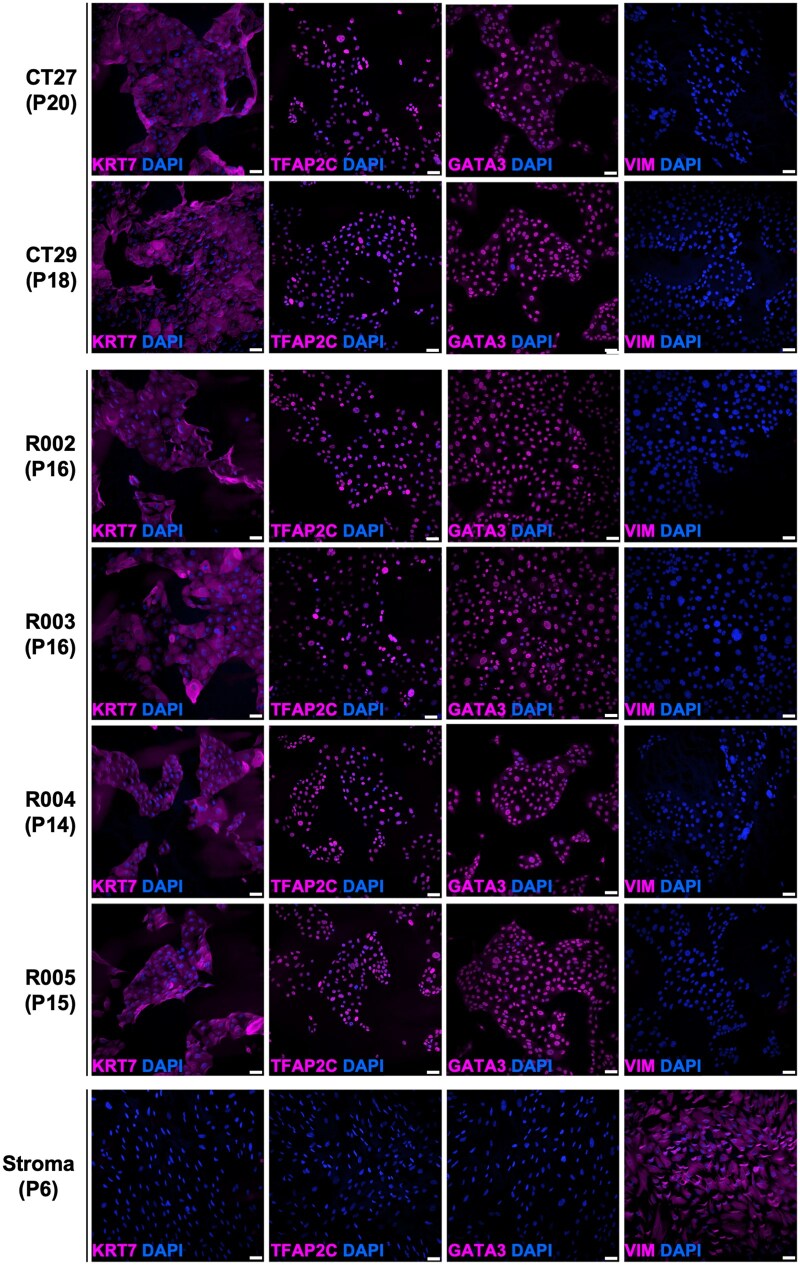
**Trophoblast stem cell marker expression in products of conception-derived trophoblast stem cells.** Products of conception (POC)-derived trophoblast stem (TS) cells express canonical trophoblast markers and lack stromal contamination. Representative immunofluorescence images of cytotrophoblast-derived (CT27, CT29), POC-derived (R002–R005), and stromal cells stained for KRT7, TFAP2C, GATA3, and VIM. Nuclei were counterstained with DAPI. Scale bar, 50 µm. Experiments were performed in triplicate for each marker and cell line. POC, products of conception; TS, trophoblast stem; KRT7, cytokeratin 7; TFAP2C, transcription factor AP-2 gamma; GATA3, GATA binding protein 3; VIM, vimentin; DAPI, 4′,6-diamidino-2-phenylindole.

Cell type-specific markers KRT7, TFAP2C, and GATA3 were evaluated at the protein level by immunocytochemistry. All TS cells evaluated showed positive cytoplasmic staining of epithelial cell marker KRT7 (Fig. 3 and Supplementary Fig. S4). Similarly, the nuclei of TS cells stained positively for TFAP2C and GATA3 (Fig. 3 and Supplementary Fig. S5). KRT7, TFAP2C, and GATA3 signals were absent in stromal cells. To rule out the presence of stromal cells in TS cell cultures, we also probed cells for vimentin (VIM). VIM signal was not detected in any TS cells evaluated, but strong signal was observed in the stromal cells used as a positive control. Therefore, POC-derived TS cells expressed key trophoblast markers, and cultures did not contain stromal cells. Collectively, these findings indicate that POC-derived TS cells exhibited defining features of trophoblast identity.

### HLA class I expression was modifiable in TS cells

**Figure 4.**
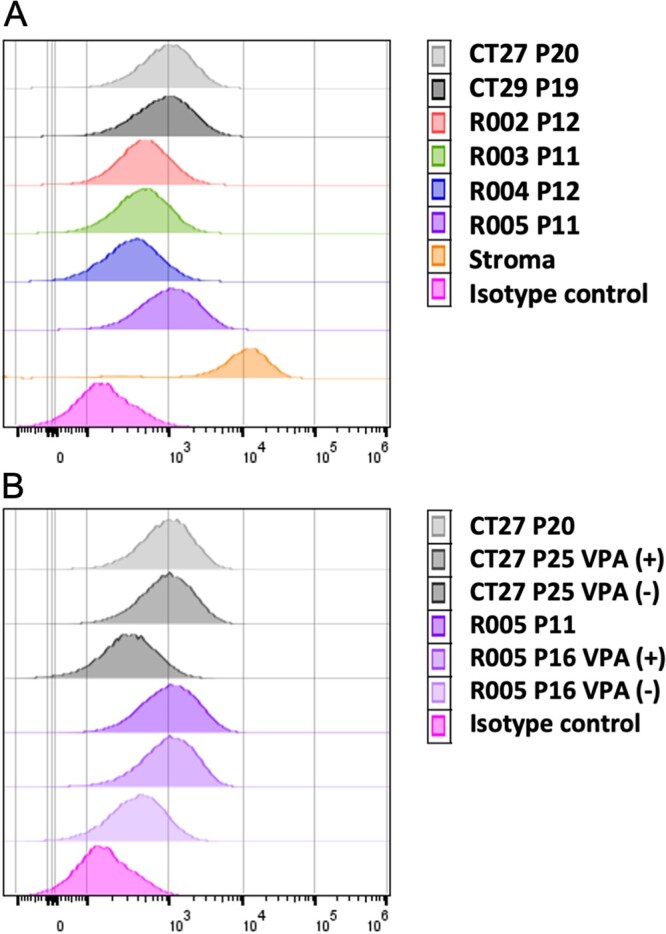
**Regulation of HLA class I expression in trophoblast stem cells.** HLA class I expression varies across TS cells and is modulated by culture conditions. (**A**) Flow cytometric analysis of HLA-ABC expression in cytotrophoblast-derived (CT27, CT29), POC-derived (R002–R005) TS cells, and stromal cells (positive control). (**B**) HLA-ABC expression in CT27 and R005 TS cells cultured with or without valproic acid (VPA) for 15 days (∼5 passages). Results are representative of three independent experiments. TS, trophoblast stem; POC, products of conception; VPA, valproic acid.

HLA Class I genes (HLA-ABC) encode cell surface proteins that present peptide antigens to T cells, a critical aspect of immune system functionality (Choo, 2007). HLA Class I molecule expression is low in primary trophoblasts (King *et al.*, 2000). Okae *et al.* (2018) previously reported low HLA-ABC expression levels (2.25%) in CT27 and CT29 TS cells at time of initial derivation and characterization. As the HLA-ABC expression profile is considered a key feature of trophoblast, we evaluated expression using flow cytometry (Fig. 4 and Supplementary Fig. S6). Stromal cells were used as a cell type positive control at 86.2% HLA-ABC positive (Fig. 4A). POC-derived TS cell lines R002, R003, and R004 expressed low levels of HLA-ABC (3.05%, 2.48%, and 1.26%, respectively; Fig. 4A and Supplementary Fig. S7). Thus, HLA-ABC expression was compatible between R002, R003, and R004 cells and those initially reported by Okae *et al.* However, HLA-ABC expression in R005 TS cells from an equivalent passage number was elevated at 21.1%. Interestingly, TS reference cell lines, CT27 and CT29 analyzed in parallel, expressed higher levels of HLA-ABC than those initially reported at 15.8% and 15.6%, respectively (Fig. 4A and Supplementary Fig. S7). Thus, we speculated that HLA-ABC expression may be modified by passaging and/or time in culture.

VPA, a potent inhibitor of histone deacetylases (HDACs) and a reagent used in TS cell culture medium to increase cell proliferation, was shown to induce HLA-ABC expression in TS cells (Soncin *et al.*, 2022). To determine whether HLA-ABC expression was impacted by the presence of VPA in the culture medium, we cultured CT27 and R005 TS cells for five passages over the course of 15 days with and without inclusion of VPA. Culturing TS cells for 15 days without VPA decreased HLA-ABC expression from 15.8% to 0.85% in CT27 TS cells, and from 21.1% to 1.52% in R005 TS cells (Fig. 4B and Supplementary Fig. S7). VPA removal did not affect cell doubling time (data not shown). These data confirm that HLA Class-I expression in TS cells was induced by the inclusion of VPA in the culture medium. Thus, HLA Class-I expression was modifiable in TS cells.

### TS cells demonstrated uniform ability to form STB spheroids

Characterization performed on the newly derived TS cells maintained in stem state culture was suggestive of successful TS cell propagation. However, the true test of a TS cell state is whether the cells have the capacity to differentiate toward the terminal trophoblast lineages, STB and EVT. Assessments of cell differentiation capacity were routinely performed after the derivation phase (> passage 10). Cell differentiation was assessed at morphological and functional levels.

**Figure 5.**
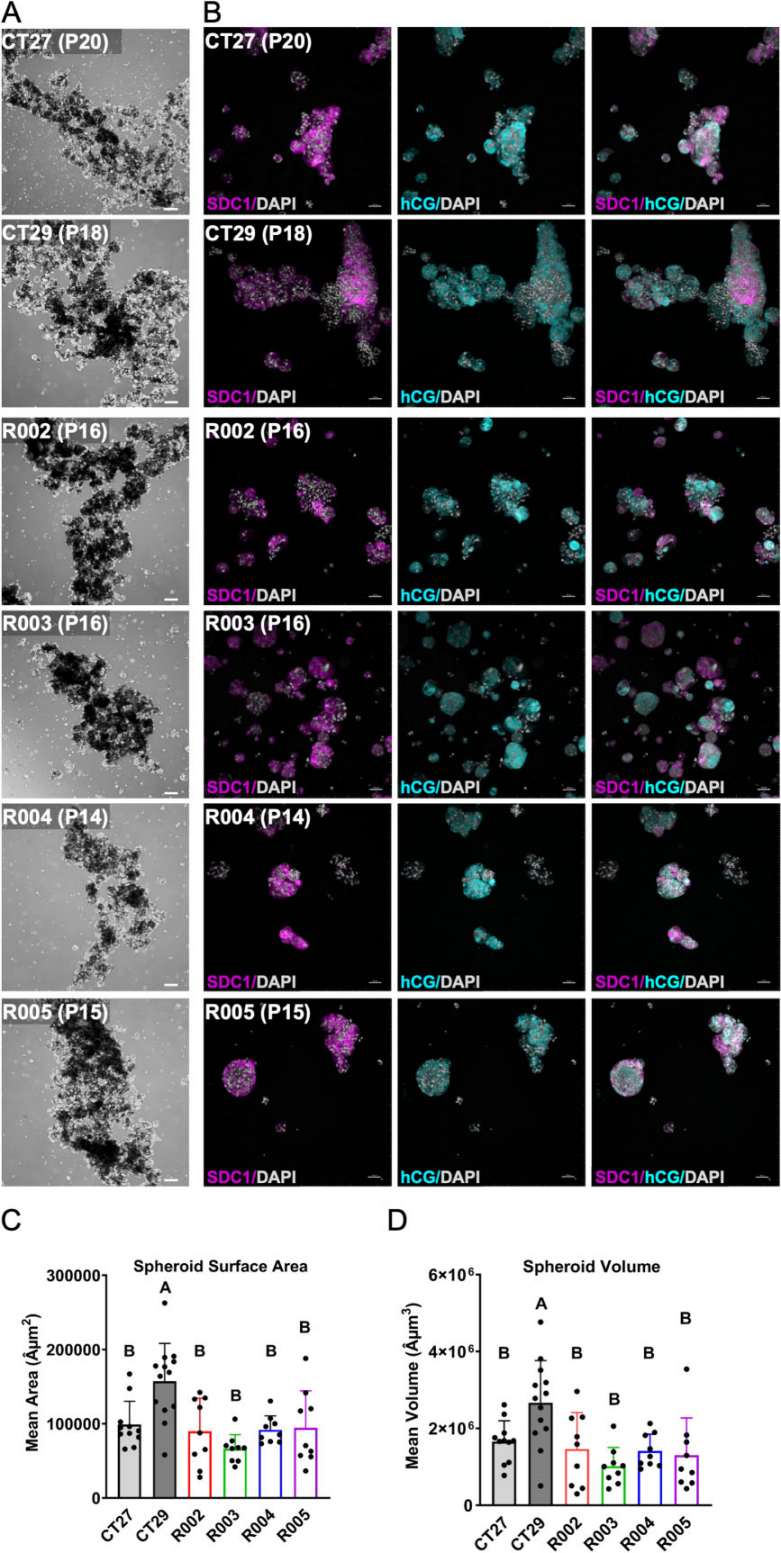
**Formation of syncytiotrophoblast spheroids by trophoblast stem cells.** TS cells from POC and cytotrophoblast sources form syncytiotrophoblast (STB) spheroids with comparable morphology. (**A**) Phase contrast images of TS cells differentiated for 6 days under 3D STB conditions. Scale bar, 250 µm. (**B**) Immunofluorescence staining for STB markers Syndecan-1 (SDC1) and human chorionic gonadotropin beta (hCGβ). Nuclei stained with DAPI. Scale bar, 100 µm. (**C, D**) Quantification of spheroid surface area and volume using 3D image analysis. Values represent mean ± SD; n ≥ 150 spheroids per sample across three independent experiments. Groups without a shared letter differ significantly (one-way ANOVA, *P* < 0.05). TS, trophoblast stem; STB, syncytiotrophoblast; SDC1, Syndecan-1; hCGβ, human chorionic gonadotropin beta.

The ability of TS cells to differentiate toward the STB lineage was assessed on Day 6 of differentiation using the previously described ST3D differentiation protocol (Okae *et al.*, 2018). STB differentiation elicited significant morphological changes in all TS cell lines. Cells formed multinucleated spheroid cell clusters that self-assemble and grew in suspension culture (Fig. 5A). STB differentiated spheroid cell clusters generated from all TS cell lines expressed STB markers Syndecan 1 (SDC1) and hCGβ (Fig. 5B). Thus, at initial assessment, POC-derived TS cells exhibited STB differentiation capacity that matched cytotrophoblast-derived TS cells. However, variability in spheroid size and structure was observed in all STB cultures and prompted more in-depth evaluation.

Based on the observation that spheroid formation appeared to be heterogeneous within a given TS cell line, and to more rigorously assess STB differentiation capacity, hundreds of independent spheroids were analyzed using a quantitative approach in Imaris (Bitplane). A combination of immunofluorescence images from SDC1 and hCGβ staining (Supplementary Fig. S1) was provided as input to quantify the surface area and volume of STB spheroid clusters. POC-derived TS cells formed spheroids of similar size to CT27 cytotrophoblast-derived TS cells (Fig. 5C and D). No differences in spheroid surface area (Fig. 5C) or volume (Fig. 5D) were observed. However, CT29 TS cells formed larger spheroids on average. Spheroids generated from CT29 TS cells had significantly increased surface area and volume compared with all other TS cell lines (*P *< 0.05; Fig. 5C and D). Measures of mean spheroid surface area and mean spheroid volume negatively correlated with total spheroid counts (Supplementary Fig. S8). The correlations were significant in spheroids from POC-derived TS cells, but not cytotrophoblast-derived TS cells (Supplementary Fig. S8). Overall, TS cells exhibited similar STB differentiation capacity in terms of their ability to syncytialize and form spheroids.

### Deficits in STB function were observed despite morphological similarities

**Figure 6.**
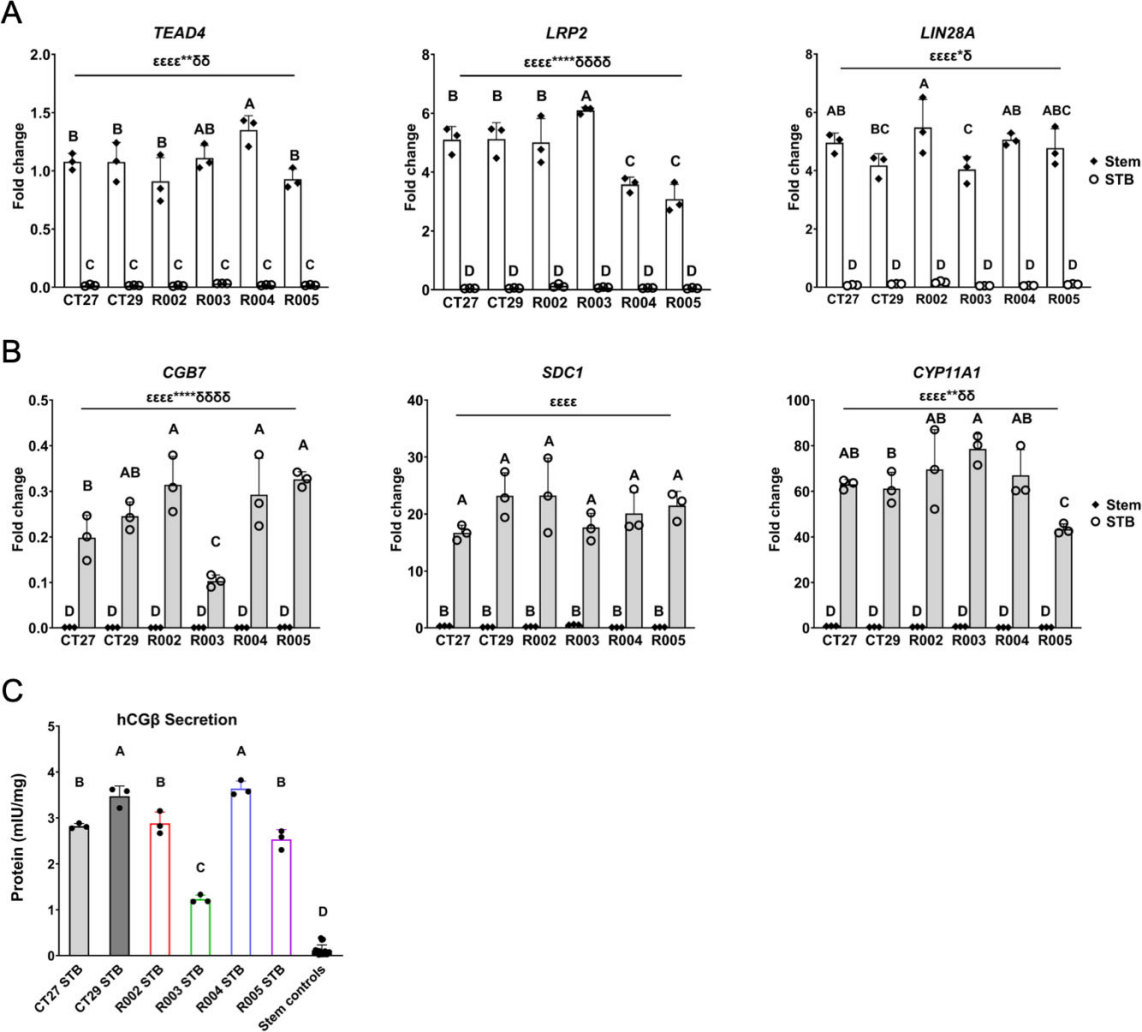
**Functional deficits in syncytiotrophoblast differentiation despite morphological similarity.** TS-derived STB cells exhibit transcriptional changes and variable hormone secretion. (**A, B**) Relative mRNA expression of stem-state genes (*TEAD4*, *LRP2*, *LIN28A*) and STB markers (*CGB7*, *SDC1*, *CYP11A1*) in stem-state (white) and STB-differentiated (gray) cells. Values normalized to *TBP*. Mean ± SD, n = 3. Statistical significance assessed by two-way ANOVA with Tukey’s *post hoc* test; ε, cell type effect; *, cell line effect; δ, interaction effect (*P* < 0.05). (**C**) hCGβ secretion measured by ELISA from culture supernatants. Values represent mean ± SD, n = 3. Groups without a shared letter differ significantly (one-way ANOVA, *P* < 0.05). TS, trophoblast stem; STB, syncytiotrophoblast; hCGβ, human chorionic gonadotropin beta; TBP, TATA-binding protein.

In addition to assessing the ability of TS cells to form syncytialized STB spheroid structures, we wanted to further evaluate the transcriptional program and functional capacity of the differentiated cells. Stem state and STB cell-specific signatures were assessed by RT-qPCR. TS cell differentiation toward the STB lineage significantly decreased the expression of stem state-specific genes, including TEA domain transcription factor 4 (*TEAD4*), LDL receptor related protein 2 (*LRP2*), and lin-28 homolog A (*LIN28A*; *P *< 0.05; Fig. 6A), although cell line-dependent variability in fold changes was observed across all three genes. Conversely, STB differentiation resulted in significant upregulation of STB-specific genes including chorionic gonadotropin beta 7 (*CGB7*), *SDC1*, and cytochrome P450 Family 11 Subfamily 1 (*CYP11A1*; *P *< 0.05; Fig. 6B). Cell line-dependent variability was observed in fold changes for *CGB7* and *CYP11A1*, while *SDC1* expression patterns were consistent (Fig. 6B). Overall, consistent and significant transcriptomic changes were observed across cell lines following STB cell differentiation.

To assess STB function at the protein level, we quantified hCGβ protein secretion by ELISA using culture medium supernatants. Supernatants from cytotrophoblast-derived CT29 and POC-derived R004 differentiated STB had the highest measured concentrations of hCGβ near 3.5 mIU/mg (Fig. 6C). Cytotrophoblast-derived CT27 and POC-derived R002 and R005 differentiated STB also secreted high levels of hCGβ near 3 mIU/mg. STB differentiated from R003 TS cells secreted significantly less hCGβ at 1 mIU/mg. Thus, most STB spheroids displayed similar functional capacity to secrete hCGβ. R003 TS cell-derived STB showed functional impairment, with significantly less hCGβ secretion (*P *< 0.05; Fig. 6C). This result is consistent with the transcriptomic difference observed in *CGB7* (Fig. 6B). Overall, POC-derived TS cells had comparable STB formation capabilities to cytotrophoblast-derived TS cells based on spheroid characteristics. However, differences were observed at the functional level, with R003 differentiated STB displaying significantly impaired hCGβ secretion capacity.

### POC-derived TS cells exhibited morphological and molecular variability in EVT cell differentiation

**Figure 7.**
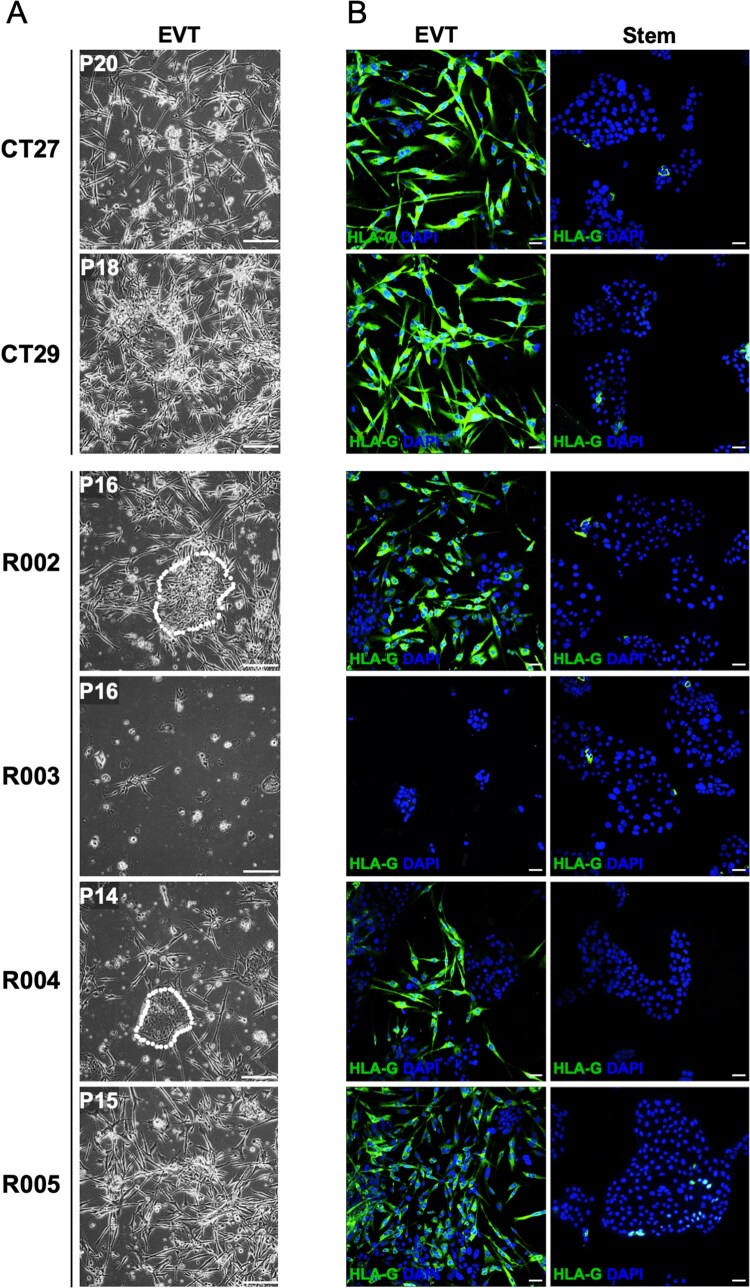
**Variability in extravillous trophoblast differentiation among trophoblast stem cells.** POC-derived TS cells exhibit variable extravillous trophoblast (EVT) differentiation capacity. Phase contrast images of TS cells following 8 days of EVT differentiation. Dashed lines indicate residual stem-state morphology. Scale bar, 250 µm. Immunofluorescence detection of HLA-G expression (green) with nuclear counterstaining (DAPI, blue). Scale bar, 50 µm. Images represent 5–8 fields per sample across three independent experiments. TS, trophoblast stem; EVT, extravillous trophoblast; HLA-G, major histocompatibility complex class I G; DAPI, 4′,6-diamidino-2-phenylindole.

**Figure 8.**
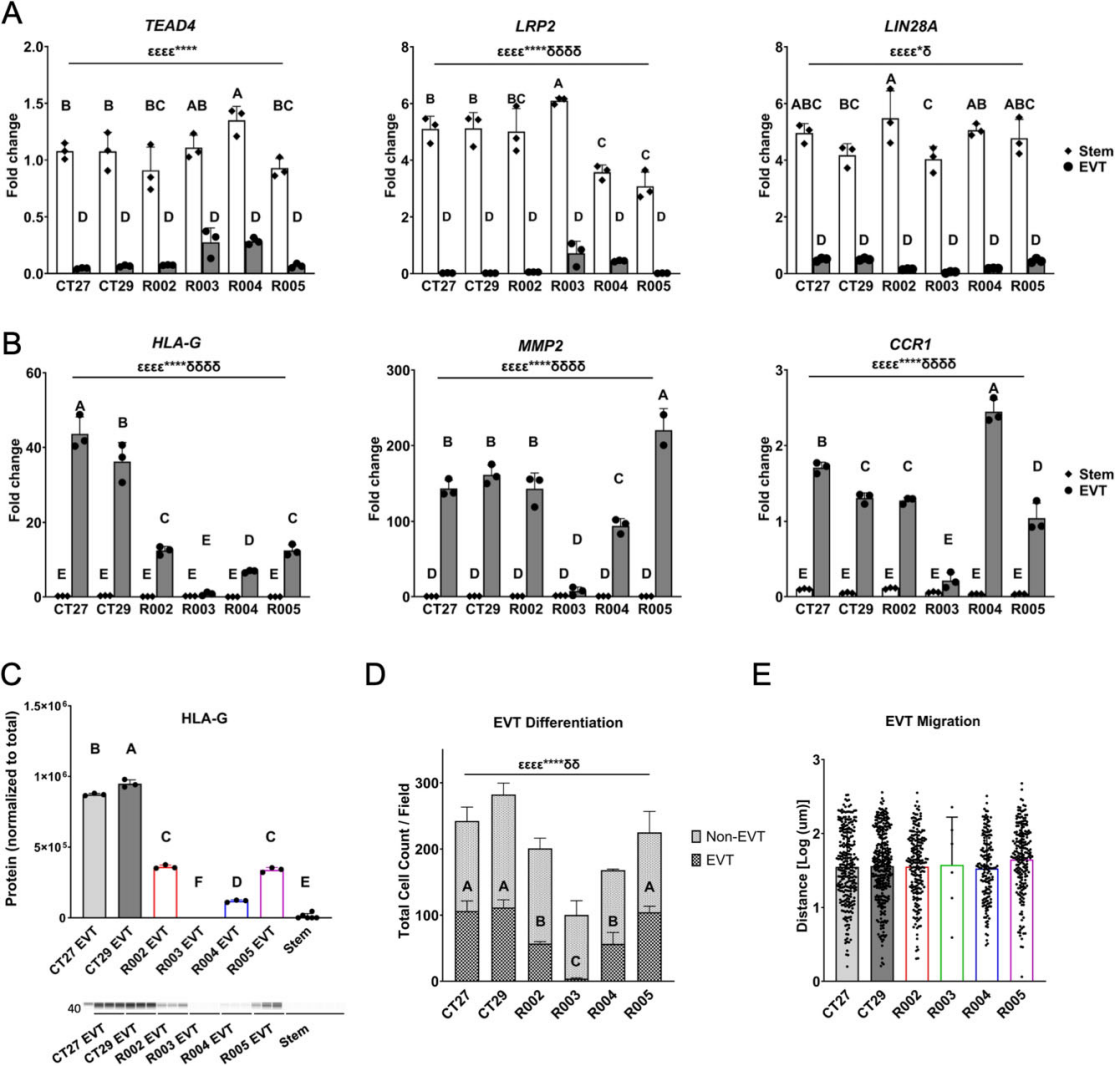
**Extravillous trophoblast differentiation efficiency is impaired in products of conception-derived trophoblast stem cells.** EVT differentiation efficiency, rather than migratory function, limits TS cell potential. (**A, B**) Expression of stem-state (*TEAD4*, *LRP2*, *LIN28A*) and EVT markers (*HLA-G*, *MMP2*, *CCR1*) following differentiation. Values normalized to *TBP*; mean ± SD, n = 3. Two-way ANOVA with Tukey’s test (*P* < 0.05). (**C**) HLA-G protein quantification normalized to total protein. Mean ± SD, n = 3; one-way ANOVA, *P* < 0.05. (**D**) Percentage of EVT cells relative to total cells after differentiation. Mean ± SD, n = 3; one-way ANOVA, *P* < 0.05. (**E**) Migration distance of EVT cells over 44 h. Statistical analysis performed using Kruskal–Wallis test with Dunn’s correction; *P* > 0.05. TS, trophoblast stem; EVT, extravillous trophoblast; TBP, TATA-binding protein.

TS cells should be evaluated for their capacity to differentiate toward the terminal trophoblast lineages, including the EVT cell lineage. Assessments of EVT cell differentiation capacity were routinely performed after the derivation phase (> passage 10). EVT cell differentiation was assessed at morphological and functional levels. The ability of TS cells to differentiate toward the EVT cell lineage was assessed on Day 8 of differentiation using the previously described EVT differentiation protocol (Okae *et al.*, 2018). Canonical features of EVT cell differentiation were observed in cytotrophoblast-derived TS cell lines CT27 and CT29. EVT-differentiated CT27 and CT29 TS cells adopted an elongated cell morphology, a stark contrast to the epithelial cobblestone colony formation in the stem state (Fig. 7A). Additionally, EVT cell differentiation in CT27 and CT29 cells resulted in upregulation of major histocompatibility complex, class I, G (HLA-G) at the RNA and protein levels (Figs 7B and 8B). EVT cell formation was variable in POC-derived TS cells (Figs 7A and 8B). EVT-differentiated TS cells from R005 adopted an elongated cell morphology and upregulated HLA-G consistent with CT27 and CT29 cell phenotypes (Fig. 7A). Following 8 days of EVT differentiation, cells from R002 and R004 samples displayed mixed cell phenotypes including patches of stem-like cells (white dashed lines) and few elongated cells (Fig. 7A). HLA-G expression was induced in the elongated cells but absent in the patches of stem-like cells (Fig. 7B). TS cells from R003 had the lowest EVT differentiation capacity, with only a few cells adopting the elongated EVT cell morphology (Fig. 7A). Consistent with the observed morphological deficits, R003 differentiated EVT cells did not express HLA-G (Fig. 7B). Thus, POC-derived TS cells exhibited varying degrees of EVT differentiation capacity.

To further confirm the morphological findings from the EVT differentiation assays, we used RT-qPCR to quantify transcriptomic signatures of TS and EVT cells. After 8 days of EVT cell differentiation, there was significant downregulation of signature stem state genes, including *TEAD4*, *LRP2*, and *LIN28A* (*P *< 0.05, Stem vs EVT; Fig. 8A). Cell line-dependent variability in fold changes was observed across all three genes. Conversely, EVT cell differentiation resulted in significant upregulation of EVT-specific genes, including *HLA-G*, matrix metalloproteinase 2 (*MMP2*), and C-C motif chemokine receptor 1 (*CCR1*; Fig. 8B). Cytotrophoblast-derived TS cells displayed consistent upregulation of *HLA-G*, *MMP2*, and *CCR1* transcripts. However, upregulation of these EVT signature genes was variable across POC-derived cell lines (*P *< 0.05; Stem vs EVT; Fig. 8B). EVT cells derived from R003 TS cells showed a near complete lack of EVT transcript induction. This finding was consistent with the morphological phenotypes observed in this sample (Fig. 8B; E vs A–D, *P *< 0.05 in *HLA-G* and *CCR1*, D vs A–C in *MMP2*, *P *< 0.05). EVT cells from R002, R004, and R005 cell lines also exhibited reduced *HLA-G* mRNA expression (Fig. 8B). This observation was maintained at the protein level, as EVT cells from R002, R003, R004, and R005 cell lines expressed significantly lower levels of HLA-G (*P *< 0.05; Fig. 8C). POC-derived TS cells displayed variable EVT cell formation capacity based on cell morphology, transcriptomic, and HLA-G expression characteristics. Deficits were prominent in R003 cells, which exhibited a complete inability to form the EVT cell lineage.

### EVT cell differentiation efficiency, not function, limited POC-derived TS cell potential

EVT cells invade the maternal compartment to facilitate spiral artery remodeling during pregnancy. To complement the assessment of EVT cell formation in POC-derived TS cells, we quantitatively assessed EVT cell function using migration assays. Cell migration was captured by live cell imaging over 2 days (∼44 h) following EVT cell re-seeding on Day 6 of differentiation. A machine learning model was trained within the Imaris software (Bitplane) to distinguish elongated EVT cells from non-differentiated cells (Supplementary Fig. S2). The ability to distinguish cell subtypes enabled quantitation of the percentage of single, migratory EVT cells out of the total number of cells from Days 6 to 8 of EVT cell differentiation. This approach also provided the ability to track migration specifically in EVT-differentiated cells. Results from the machine learning model supported the previous morphological observations that R002, R003, and R004 produced fewer EVT cells with numbers close to zero in R003 cells (*P *< 0.05; Fig. 8D). Total cell number was also reduced in R002, R003, and R004 samples despite equivalent cell numbers plated at the start of EVT differentiation (Fig. 8D). Total cell number and percentage of EVT-differentiated cells were comparable between R005, CT27, and CT29 cell lines (Fig. 8D). These findings indicated that R002, R003, and R004 cells had a markedly impaired capacity for EVT differentiation.

To further evaluate EVT function, we quantified the distance individual EVT cells moved across a 44-h period of differentiation from Days 6 through 8. The number of cells tracked, a proxy measure of differentiated cells, was different between cell lines (Fig. 8D and E). The highest numbers of EVT cells were tracked in CT27 and CT29 cell lines with slightly fewer cells tracked in R002, R004, and R005 cell lines. Consistent with the significant reduction in differentiated cells in sample R003, very few cells were tracked across the migration assay for this cell line. Despite differences in cell numbers, no significant differences in migration distance were observed between the cell lines during this period (*P *> 0.05; Fig. 8E). This observation suggested that if TS cells can differentiate into EVT cells, they maintain functional migratory capacity. Taken together, these findings show that TS cells derived from POC following RPL have impaired EVT differentiation capacity to variable degrees. Case R003 had almost completely diminished EVT differentiation capacity. Interestingly, the percentage of total TS cells that differentiated into EVT was the limiting factor. Once cells differentiated, functional deficits in migratory abilities were not evident.

Together, these data reveal marked heterogeneity across POC-derived TS cell lines, with R003 exhibiting the most pronounced defects in EVT differentiation and proliferation, while R005 most closely resembled cytotrophoblast-derived lines. To determine whether these phenotypic differences were associated with distinct molecular programs, we next performed RNA-seq on all four POC-derived TS cell lines and two cytotrophoblast-derived TS cell lines.

### Distinct transcriptional and pathway signatures distinguish RPL-derived TS cell lines by karyotype

**Figure 9.**
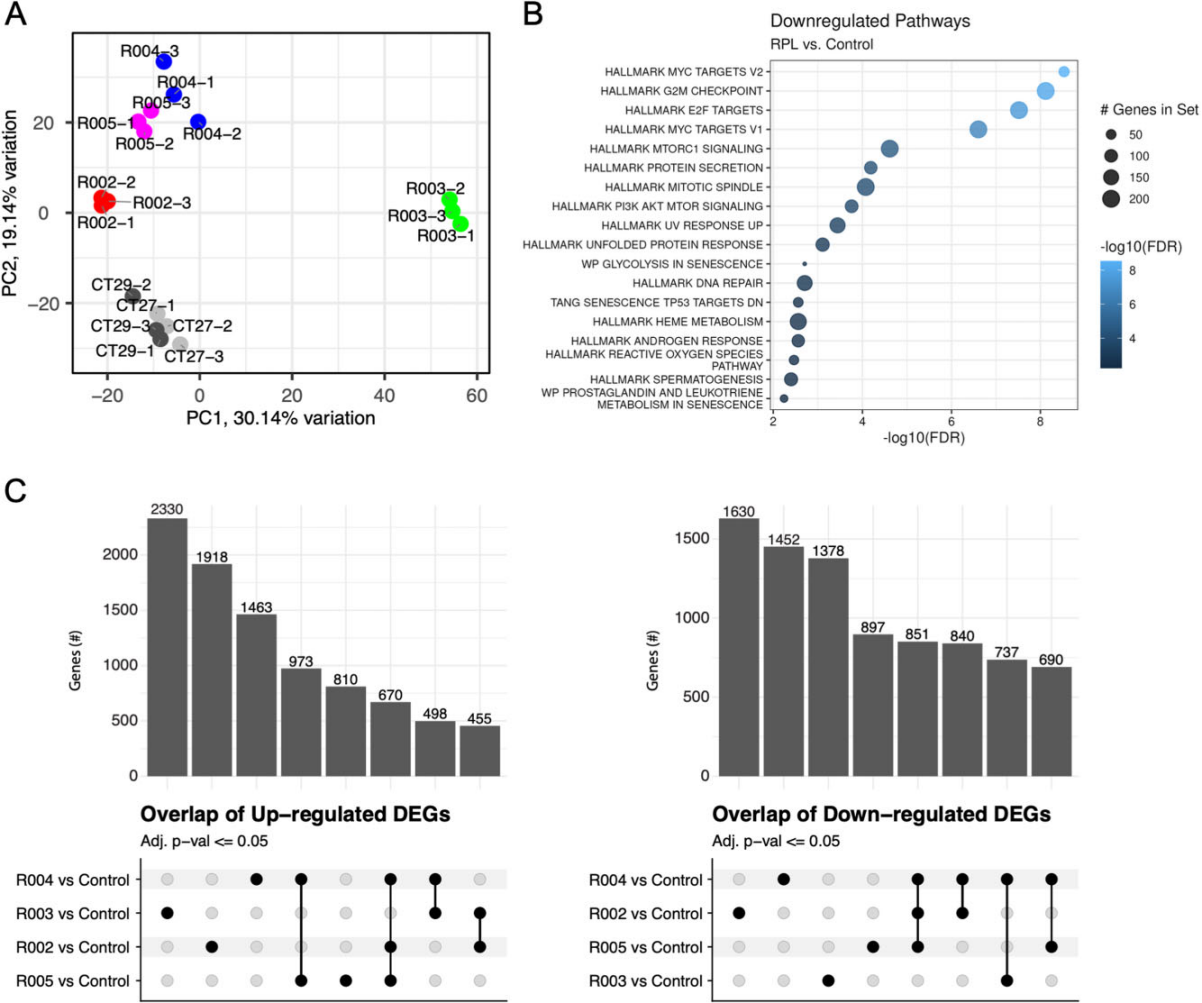
**Transcriptional profiling reveals pathway enrichment in trophoblast stem cells derived from recurrent pregnancy loss.** RNA sequencing reveals transcriptional differences between control and POC-derived TS cells. (**A**) Principal component analysis showing clustering of cytotrophoblast-derived (CT27, CT29) and POC-derived TS cell lines. Each point represents a biological replicate (n = 3 per cell line). (**B**) Gene set enrichment analysis of downregulated pathways. (**C**) UpSet plot showing overlap of differentially expressed genes across comparisons (adjusted *P* < 0.05). TS, trophoblast stem; POC, products of conception; RNA-seq, RNA sequencing; PCA, principal component analysis.

To define global transcriptional relationships among TS cell lines, we performed principal component analysis (PCA) on RNA-seq data from all lines maintained in a stem, proliferative state. Cytotrophoblast-derived TS cell lines (CT27/CT29) clustered tightly, indicating high transcriptional similarity and supporting their use as controls (Fig. 9A). Similarly, RPL POC–derived TS cell lines with normal karyotypes (R004 and R005) clustered together but segregated from cytotrophoblast controls, suggesting a shared yet distinct transcriptional program associated with pregnancy loss. In contrast, karyotypically abnormal RPL POC-derived TS cell lines (R002 and R003) showed marked divergence. R003 was the most pronounced outlier along the first two principal components, while R002 displayed intermediate separation from both control and normal-karyotype RPL clusters. These findings indicate that karyotypic abnormalities are a major source of transcriptional heterogeneity among TS cell lines.

To define pathways underlying these differences, we performed differential expression and pathway enrichment analyses across all TS cell lines. Comparing all RPL POC-derived cell lines with controls (CT27/CT29), significant enrichment was observed only among downregulated differentially expressed genes (DEGs; Fig. 9B). DEGs included MYC targets, G2/M checkpoint, E2F targets, MTOR signaling, mitotic spindle, and senescence-associated pathways. Because RPL lines showed divergent transcriptomes by PCA and included a mix of karyotypes, we next assessed overlap of up- and downregulated DEGs in pairwise comparisons of each RPL line (R002–R005) versus controls. Upset plots highlight the shared and unique DEGs across comparisons (Fig. 9C). The bars quantify the number of genes per intersection, while the accompanying filled circles indicate which comparisons contribute to each overlapping gene set. The greatest number of up- and downregulated DEGs was found among individual comparisons of R003, R002, and R004 cells versus Control (Fig. 9C). However, some commonalities in DEGs were identified across comparisons, such as the 670 upregulated DEGs identified as common between R004, R002, and R005 when individually compared to the control cell lines. To facilitate biological interpretation of these gene-level changes and identify shared versus cell line-specific molecular programs, we next performed pathway enrichment analyses across all comparisons.

**Figure 10.**
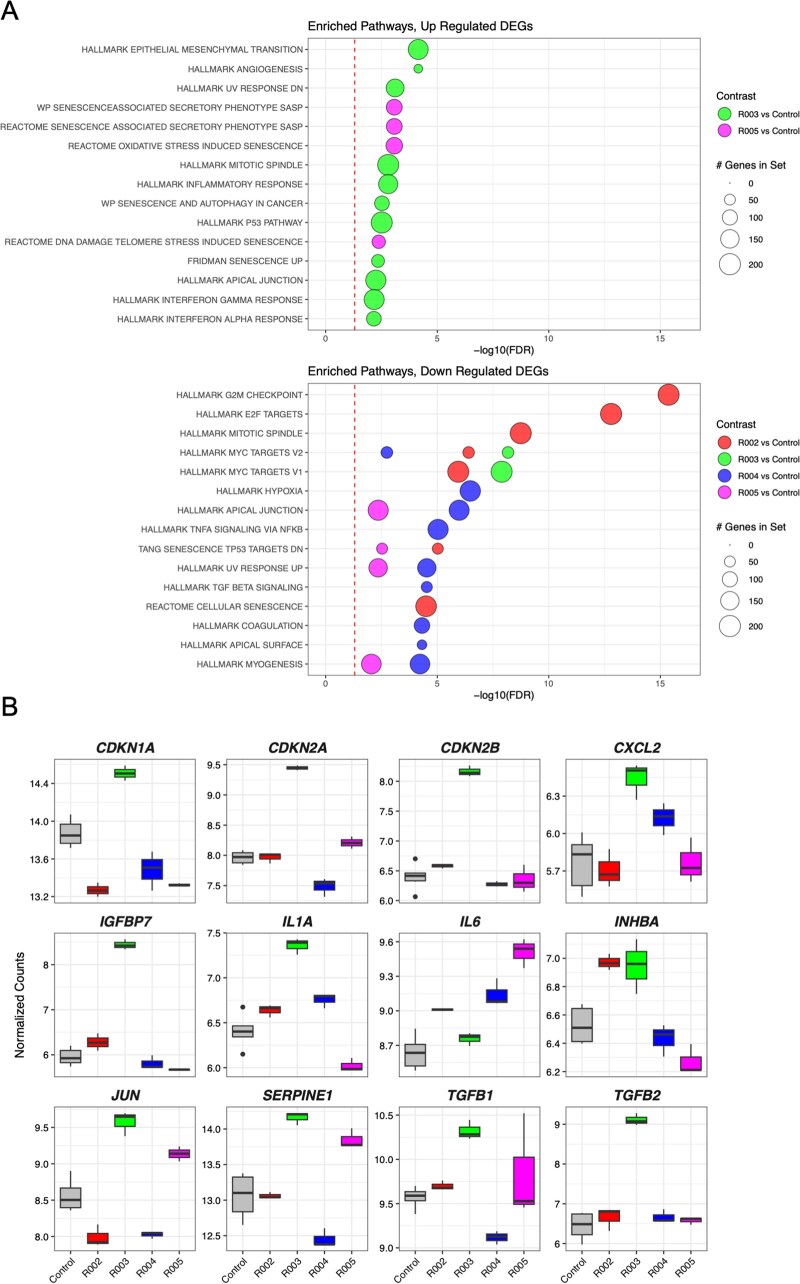
**Pathway heterogeneity across individual trophoblast stem cell lines derived from products of conception.** Distinct pathway signatures differentiate individual TS cell lines. (**A**) Gene set enrichment analysis of upregulated (top) and downregulated (bottom) pathways across comparisons (log2-fold change ≥ 1, adjusted *P* < 0.05). (**B**) Expression levels of senescence-associated genes (*CDKN1A*, *CDKN2A*, *CDKN2B*, *CXCL2*, *IGFBP7*, *IL1A*, *IL6*, *INHBA*, *JUN*, *SERPINE1*, *TGFB1*, *TGFB2*) across TS cell lines. TS, trophoblast stem; POC, products of conception; DEGs, differentially expressed genes.

Fifteen pathways were significantly enriched based on upregulated DEGs across TS cell line comparisons; however, significant pathway enrichment was confined to the R003 versus Control and R005 versus Control comparisons (Fig. 10A). R003 TS cells exhibited a distinct transcriptional profile characterized by enrichment of epithelial–mesenchymal transition, mitotic spindle, inflammatory response, and senescence-associated pathways (Fig. 10A). Consistent with these transcriptional signatures, R003 cells displayed senescent-like morphology (flattened, enlarged; Fig. 2A) and increased expression of canonical senescence regulators, including *CDKN1A*, *CDKN2A*, and *CDKN2B* and senescence-associated secretory phenotype (SASP)-related genes such as *SERPINE1* (PAI1), *TGFB1-2*, *CXCL2*, *IGFBP7*, *INHBA*, and *IL1A*, a key upstream regulator of SASP induction (Fig. 10B). R005 TS cells also showed significant enrichment of senescence-associated, stress-responsive SASP programs. Specifically, R005 cells exhibited coordinated upregulation of SASP-related genes, including *SERPINE1* (PAI1), *TGFB1*, *JUN*, and *IL6* (Fig. 10B). These findings suggest that distinct, cell line-specific senescence programs operate within abnormal- and normal-karyotype RPL POC-derived TS cells, contributing to transcriptional heterogeneity beyond pregnancy loss status.

In contrast to the more limited enrichment observed among upregulated genes, analysis of downregulated DEGs further distinguished TS cell line phenotypes (Fig. 10A). Pathways were identified based on downregulated genes (with the top 15 shown) across all RPL-derived lines relative to controls. R002 showed pronounced repression of core cell cycle pathways, including G2/M checkpoint, E2F targets, and mitotic spindle, which is consistent with its higher doubling time (Fig. 1C). Several POC-derived TS cell lines (R002, R003, and R004) also exhibited downregulation of MYC targets (Fig. 10B). Additional affected pathways included hypoxia, apical junction, NFκB, TP53, TGFβ signaling, and senescence.

Overall, RPL POC-derived TS cell lines segregate by karyotype state, with abnormal-karyotype RPL lines exhibiting heterogeneous transcriptional and pathway signatures. These data identify karyotypic status as a key determinant of TS cell line identity and functional state.

## Discussion

In this study, we established patient-specific TS cell lines from POC tissue following RPL and performed integrated phenotypic and functional characterization across stem and differentiation states. Our first major finding is that these lines exhibited pronounced functional heterogeneity that was not always evident from stem-state markers or morphology alone (R003 the exception), despite uniformly meeting established criteria for trophoblast identity. Variability was most apparent during lineage commitment, with EVT differentiation efficiency emerging as the primary axis distinguishing patient-specific tissue-derived cells. Our second major finding underscores the importance of incorporating TS cell lines that encompass both normal and abnormal cytogenetic backgrounds. Given the high prevalence of chromosomal abnormalities in early pregnancy loss, POC-derived TS cells offer a unique opportunity to examine naturally occurring genetic perturbations that are otherwise underrepresented in placental research. We propose that establishing patient-specific POC-derived TS cells will reveal mechanistic insights into trophoblast dysfunction relevant to early placental failure and RPL.

TS cells offer a powerful platform for studying early placental development, as they can be maintained in a proliferative stem-like state or directed toward either STB or EVT cell lineages. The multipotent capacity of TS cells has enabled the identification of key regulatory pathways governing placental development and trophoblast lineage decisions (Bhattacharya *et al.*, 2020; Jaju Bhattad *et al.*, 2020; Saha *et al.*, 2020; Hornbachner *et al.*, 2021; Muto *et al.*, 2021; Perez-Garcia *et al.*, 2021; Varberg *et al.*, 2021, 2023; Toh *et al.*, 2024; Oike *et al.*, 2025). More recently, TS cells derived from POC have demonstrated value for uncovering molecular mechanisms underlying trophoblast lineage development in failed pregnancies (Saha *et al.*, 2020; Kumar *et al.*, 2024). However, the extent to which TS cells faithfully recapitulate early STB lineage establishment remains an area of active investigation, as some evidence suggests that TS cells may preferentially represent EVT progenitors (Shannon *et al.*, 2024; Morey *et al.*, 2025). In this context, evaluating differentiation toward both STB and EVT lineages provides a critical framework for assessing trophoblast competence and reveals whether preserved stem-state identity translates into functional multipotency.

Although all four RPL POC-derived TS cell lines formed morphologically comparable STB spheroids, functional readouts revealed cell line-specific differences in hormone secretion. This dissociation between morphology and function underscores the importance of incorporating functional benchmarks when evaluating trophoblast differentiation potential. EVT differentiation capacity varied markedly across RPL POC-derived TS cell lines, with the primary limitation arising at the level of differentiation efficiency rather than migratory function. Notably, once TS cell adopted an EVT identity, their migratory behavior was largely preserved, suggesting that impaired lineage commitment, not intrinsic functional defects, may underlie the deficits observed. This distinction between preserved trophoblast identity and divergent differentiation potential highlights a broader limitation in current *in vitro* placental modeling approaches, which often rely on static phenotypic benchmarks to define cell state. Although we quantified cell migration over time at the single-cell level, our study did not include invasion assays. Nevertheless, our observations underscore the need to integrate functional assessments into TS cell validation frameworks, particularly when modeling early placental pathologies such as RPL, where subtle impairments in lineage establishment may have profound developmental consequences.

The inclusion of cytogenetically abnormal patient-derived TS cell lines expands the experimental toolkit available for studying RPL pathogenesis. Notably, the karyotypically abnormal R003 TS cells fulfill most established criteria for human TS cell identity, including trophoblast-specific molecular markers and ELF5 promoter hypomethylation, supporting their classification as bona fide TS cells (Lee *et al.*, 2016). Despite this preserved stem-state identity, R003 cells exhibited atypical features, including altered morphology, prolonged doubling time, and impaired differentiation capacity, indicating a functional deficit. In this context, the R003 TS line offers a patient-derived *in vitro* system in which disease- and environment-associated constraints on proliferation and differentiation in the context of RPL can be explored mechanistically.

Variation in cell proliferation emerged as a phenotype associated with both STB and EVT cell differentiation efficiency. Differences in cell number and growth dynamics across differentiation contexts suggest that proliferation capacity may influence the efficiency with which TS cells establish differentiated trophoblast lineages. Consistent with our findings, connections between cell cycle regulation and cell differentiation have been noted across multiple stem cell systems (Hong *et al.*, 2009; Ruiz *et al.*, 2011). While this relationship is not conceptually novel in the broader stem cell field, its manifestation in patient-derived TS cells highlights its potential relevance in the context of placental pathology. Although direct studies in human TS cells remain limited, available evidence indicates that trophoblast proliferation and differentiation are tightly coordinated, and that disruptions in proliferative capacity are associated with impaired lineage specification (Okae *et al.*, 2018). We propose that extended cell-cycle duration may constrain lineage commitment, potentially by limiting progenitor expansion or the temporal window for integration of differentiation cues. Importantly, this relationship is currently hypothesis-generating, and further studies will be required to determine whether proliferation actively regulates trophoblast differentiation or instead reflects broader constraints on TS cell competence in distinct pregnancy pathologies, for which these TS cell lines may serve as valuable experimental models.

Additionally, these patient-specific TS cell lines could serve as disease-relevant models to explore cellular senescence as a potential mechanism contributing to RPL pathogenesis (Chen *et al.*, 2026). Cellular senescence is characterized by stable cell-cycle arrest and acquisition of SASP (Munoz-Espin and Serrano, 2014). Transcriptomic profiling revealed dysregulation of senescence, cell cycle, MYC targets, and p53-associated pathways in subsets of RPL TS cell lines relative to cytotrophoblast-derived TS lines. Consistent with an activated senescence-associated program, some RPL TS cells exhibit upregulation of canonical SASP-related genes, including factors previously linked to placental pathology such as *SERPINE1* (PAI1) and *INHBA* (Nonn *et al.*, 2025). This pattern is suggestive of an early or partial senescent state rather than terminal senescence. Further, downregulation of MYC target genes in R002 and R003 cells is consistent with MYC’s established role in restraining premature senescence in trophoblast cells (Singh *et al.*, 2023). However, not all cell lines showed coordinated alterations in both MYC and senescence-associated pathways, suggesting that these programs may be at least partially uncoupled.

Emerging evidence implicates trophoblast senescence in placental dysfunction during early pregnancy, including associations with early-onset preeclampsia and adverse maternal–fetal interface remodeling (Nonn *et al.*, 2025; Sun *et al.*, 2026). The observed senescence-associated signature therefore links these patient-derived TS cells to clinically relevant placental pathophysiology. Importantly, senescence programs remain largely unexplored in patient-derived TS cells, particularly in the context of RPL, highlighting the potential utility of these cell lines for identifying intrinsic cellular states that may predispose trophoblast cells to premature functional decline and contribute to RPL.

This study is limited by the small number of TS cell lines and the inherent clinical heterogeneity of RPL. However, these constraints underscore the need for expanded phenotype-stratified collections of patient-derived TS cell lines that enable comparative analyses across shared clinical features. Establishing more TS cell lines representing diverse cytogenetic and clinical contexts will be essential for identifying convergent molecular pathways that contribute to placental dysfunction in RPL and other pregnancy disorders.

Using a clinically accessible tissue source, we established patient-specific TS cell lines that retain trophoblast identity yet exhibit meaningful functional heterogeneity across differentiation states. These findings reinforce the importance of functional validation, rather than reliance on morphology or stem-state markers alone, when evaluating TS cell competence and disease relevance. By integrating phenotypic, functional, and molecular assessments across multiple patient-derived lines, this study provides a framework for leveraging patient-specific placental cells to interrogate mechanisms underlying early pregnancy failure. Although individual cell lines capture distinct aspects of trophoblast dysfunction, comparative analyses across diverse TS cell lines offer a powerful strategy to identify convergent pathways that may underlie RPL and related placental disorders.

## Supplementary Material

gaag033_Supplementary_Data

## Data Availability

Raw sequencing files and the processed gene counts matrix have been deposited in the NCBI Gene Expression Omnibus (GSE325197). Code used for all RNAseq analysis and visualizations is available at www.github.com/jmvarberg/Fu_et_al_2026. Materials generated by this study are available from the corresponding authors upon written request. Requests outlining the intended use of the materials for academic research will be evaluated for compliance with institutional policies and applicable ethical requirements.

## Author Biography

**Dr. Kaela Varberg:** Kaela Varberg, PhD is an Independent Investigator in the Fetal Health Center at Children’s Mercy in Kansas City, KS, USA. She is also jointly appointed as an Assistant Professor at the University of Missouri Kansas City and at the University of Kansas Medical Center. Dr Varberg is currently supported by a pathway to independence R00 award from the NICHD to study molecular mechanisms driving trophoblast cell lineage development. Her research program combines the use of patient-specific *in vitro* models and epigenomic approaches to identify critical mechanisms of placental development in settings of health and disease.
