# Supplementary material for: Functional heterogeneity in trophoblast stem cells derived from recurrent pregnancy loss products of conception
Source: Mol Hum Reprod. 2026 May 29;32(2):gaag033. doi: 10.1093/molehr/gaag033 (PMC13293087; doi:10.1093/molehr/gaag033)
Supplement: gaag033_Supplementary_Data [file gaag033_supplementary_data.zip › Supplementary Material_Revision_V2.docx]

**Supplementary Information**

**Functional heterogeneity in trophoblast stem cells derived from recurrent pregnancy loss products of conception**

Qi Fu, Keisuke Kozai, Xingrao Ke, Joseph M. Varberg, Sheng Xia, Ashley S. Howard, Michael Lydic, Kristin Holoch, Courtney Marsh, Elin Grundberg, and Kaela M. Varberg

| **Content** | **Page #** |
| --- | --- |
| Supplementary Figure S1. **Data acquisition and quantitative analysis of STB spheroids.** | 2 |
| Supplementary Figure S2. **Machine learning–based classification and tracking of EVT cell morphology in time-lapse imaging.** | 3 |
| Supplementary Figure S3. **Failure of trophoblast stem (TS) cell derivation from R001 products of conception (POC).** | 4 |
| Supplementary Figure S4. **Expression of trophoblast markers in TS cells.** | 5 |
| Supplementary Figure S5. **Representative TFAP2C immunofluorescence in TS cells.** | 6 |
| Supplementary Figure S6. **Flow cytometry gating strategy for identification of HLA-ABC–positive cells.** | 7 |
| Supplementary Figure S7. **Quantification of HLA-ABC–positive TS cells.** | 8 |
| Supplementary Figure S8. **Inverse relationship between spheroid size and number in STB differentiation.**  Supplementary Table S1. **Sample Metadata**  Supplementary Table S2. **Normalized Read Counts**  *Provided as a separate .xlsx file.  Supplementary Table S3. **DESeq2 DE Results**  *Provided as a separate .xlsx file. | 9  10 |


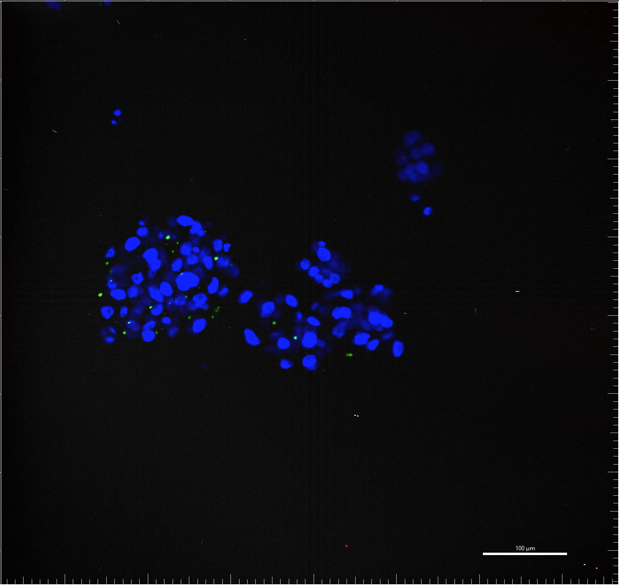


Anti-rabbit IgG/DAPI


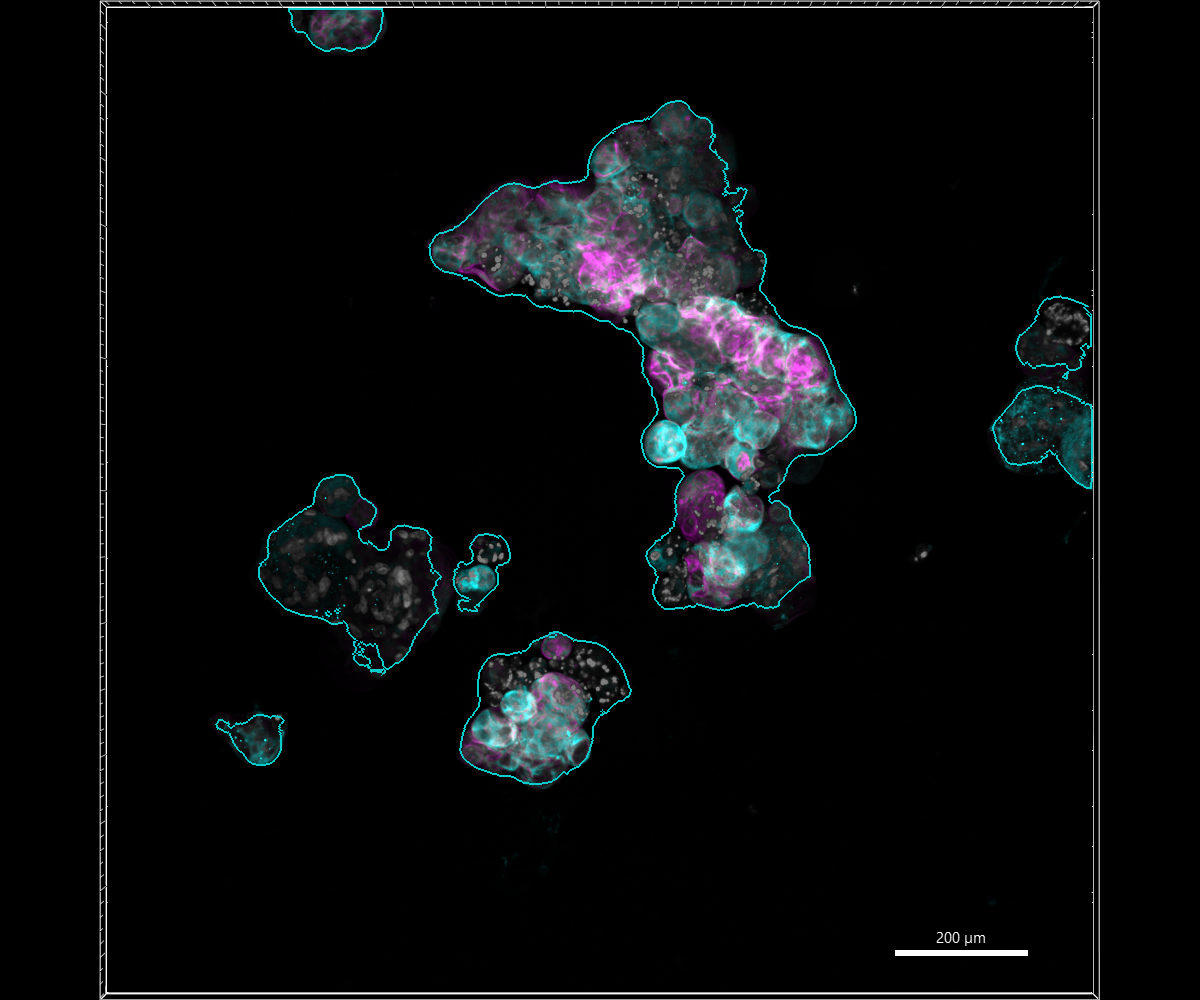

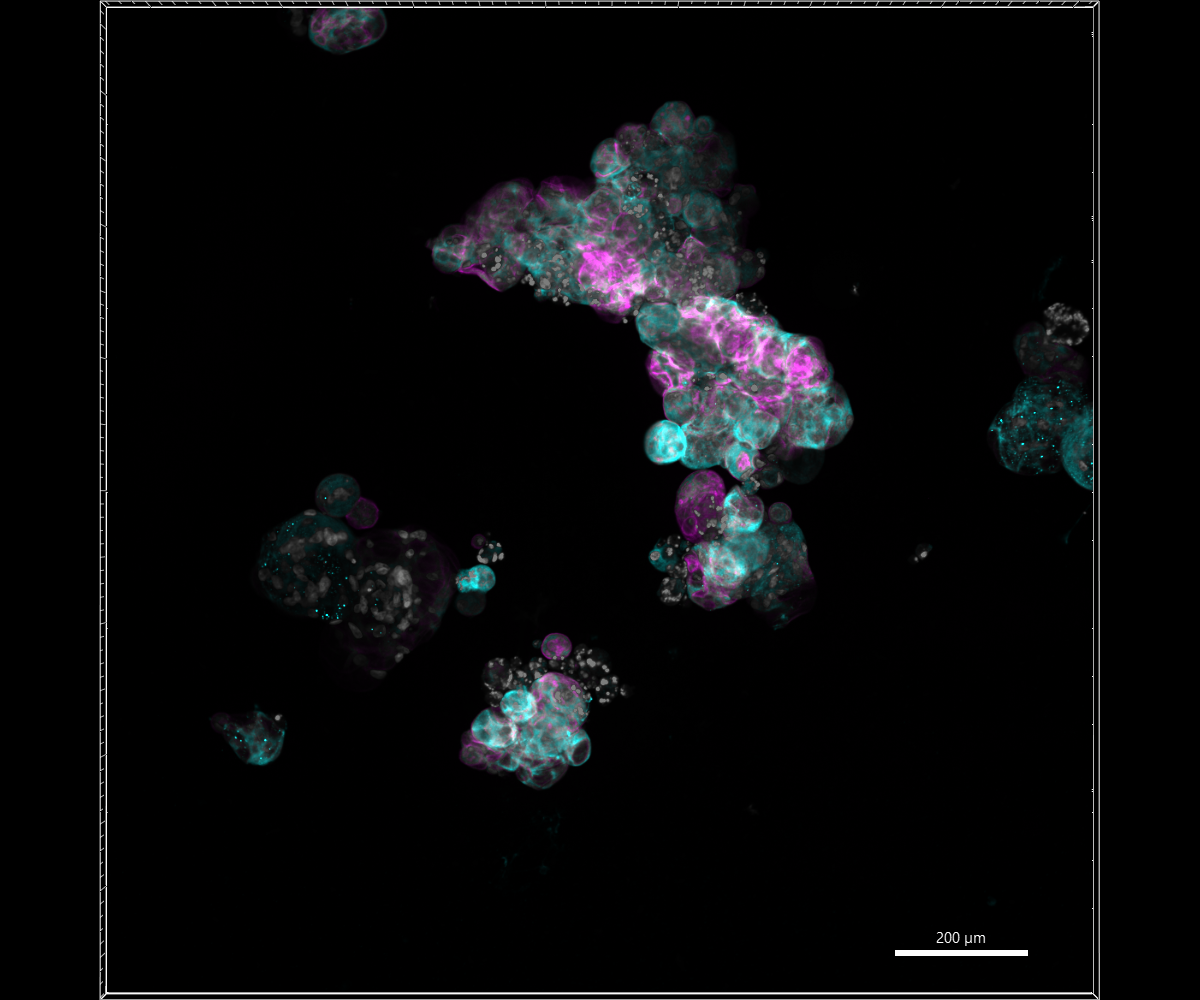

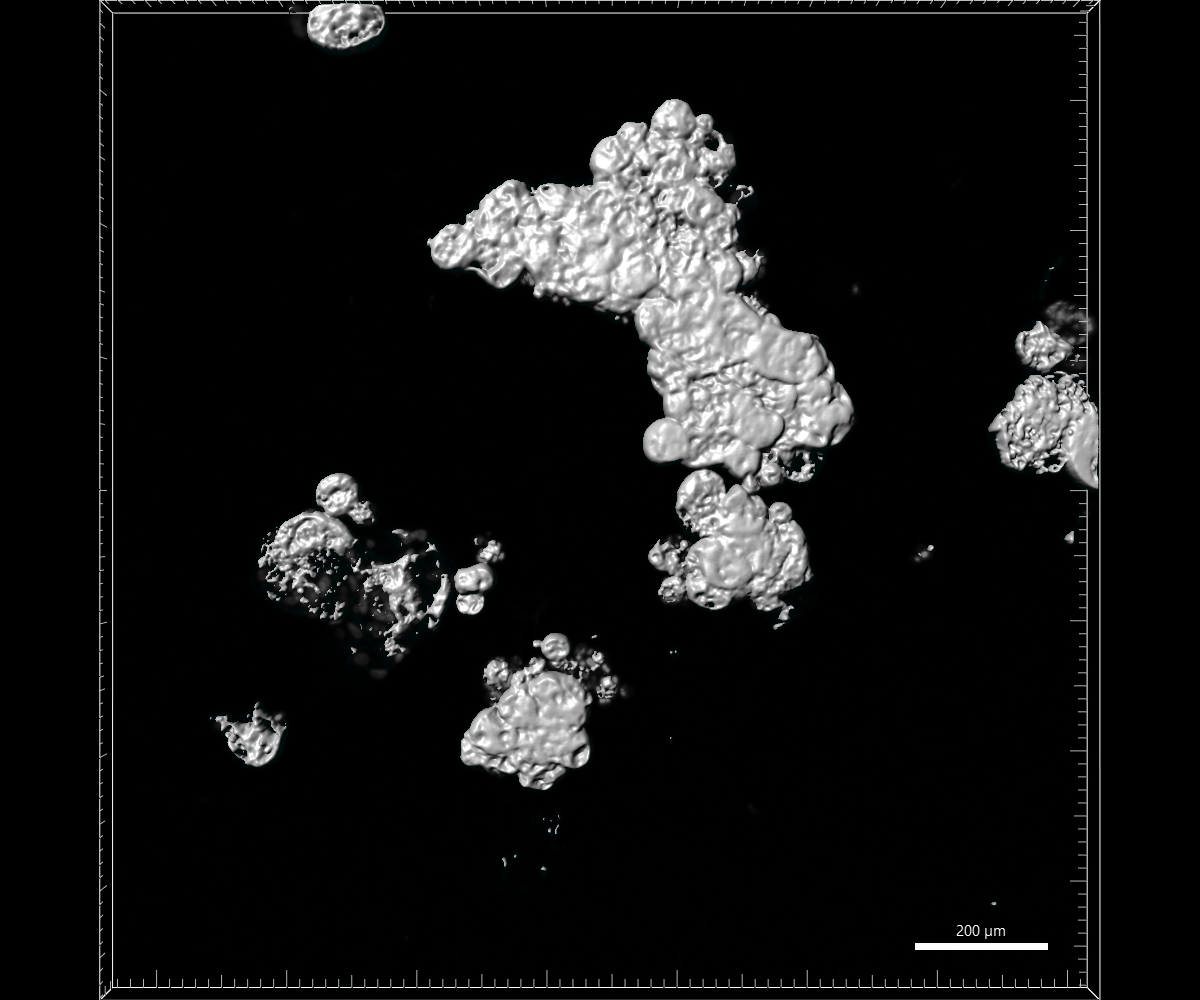


**A**

**B**

SDC1/hCG

SDC1/hCG

**Supplementary Figure S1. Data acquisition and quantitative analysis of STB spheroids.** Three-dimensional (3D) spheroid analysis was performed using Imaris® software (version 10.2.0; Bitplane, UK). Fluorescent channels included syndecan-1 (SDC1; magenta), human chorionic gonadotropin (hCG; cyan), and 4′,6-diamidino-2-phenylindole (DAPI; blue). Image files were converted to .ims format, and a machine learning–based surface segmentation algorithm was trained on a representative control sample (CT27; cytotrophoblast-derived trophoblast stem cell line) and applied to all datasets. (A) Representative images demonstrating 3D surface rendering of syncytiotrophoblast (STB) spheroids and segmentation workflow used to quantify spheroid number, surface area, and volume. (B) Negative control immunofluorescence image using secondary antibody only (anti-rabbit IgG) to confirm specificity of staining. Scale bars = 250 μm.


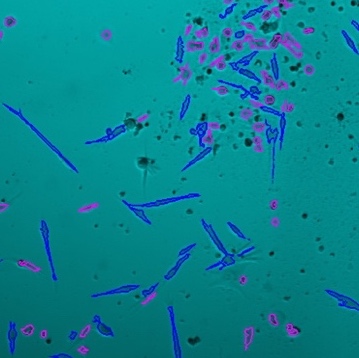

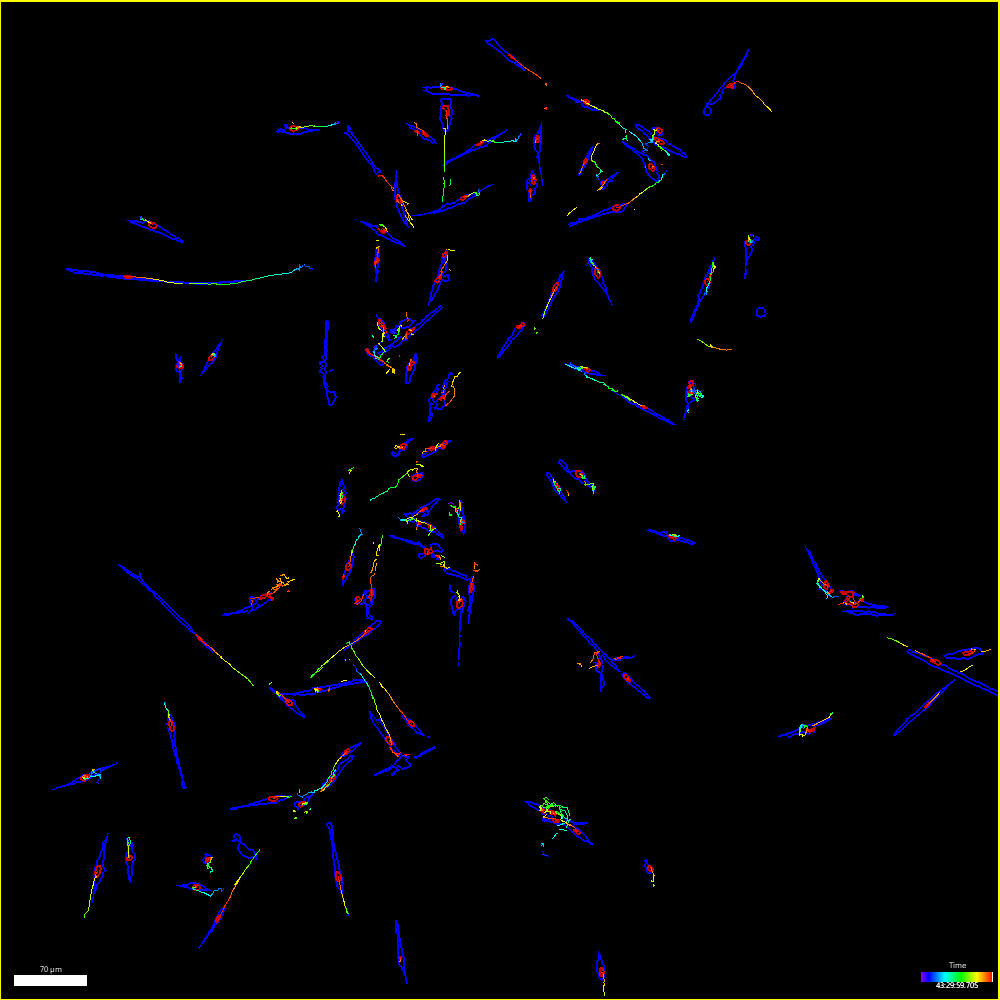

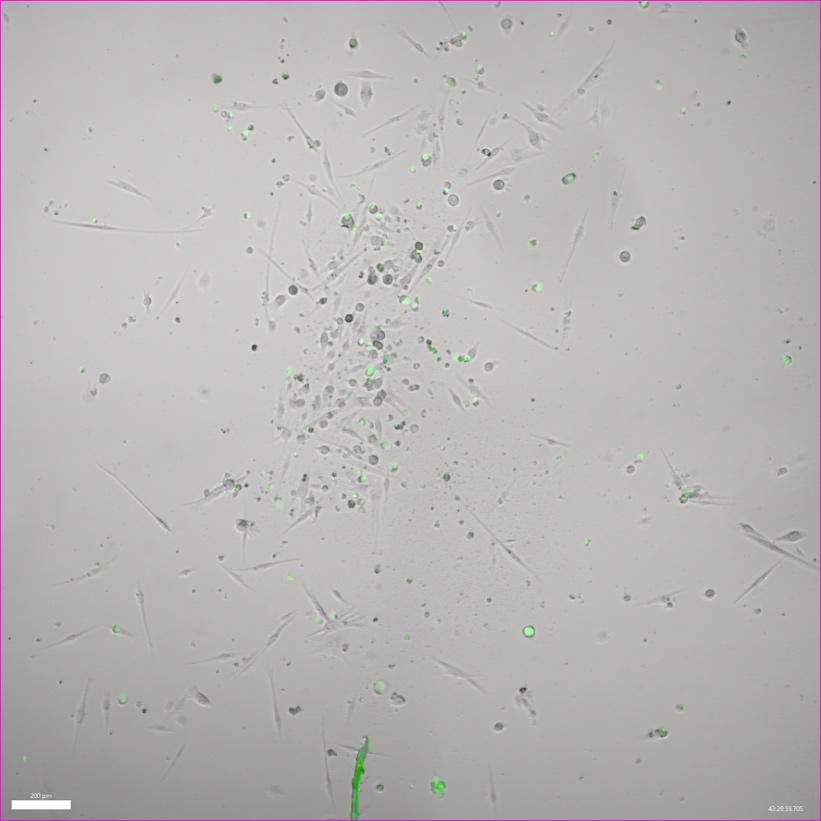


**B**

**A**

**D**


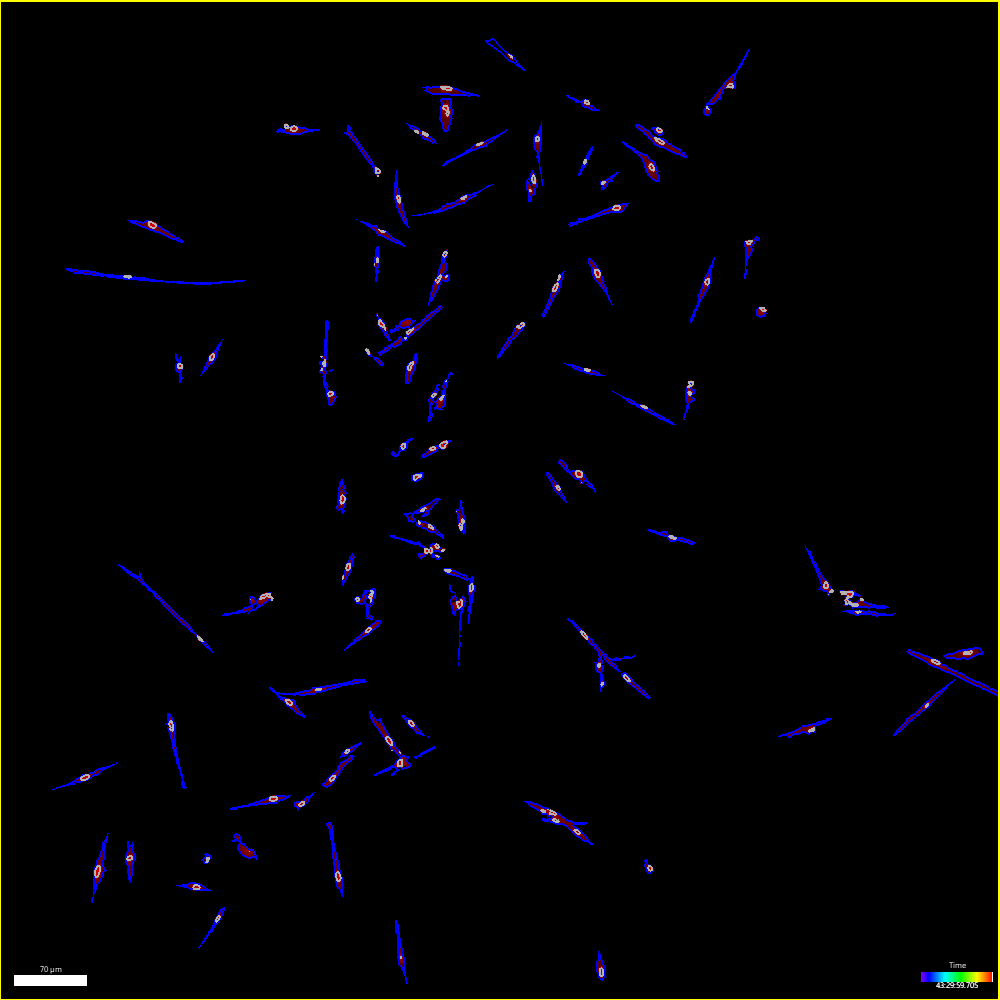


**C**

**Supplementary Figure S2. Machine learning–based classification and tracking of EVT cell morphology in time-lapse imaging.** Extravillous trophoblast (EVT) differentiation and migration were quantified using live-cell imaging and Imaris® analysis. (A) Time-lapse imaging of cells expressing green fluorescent protein (GFP) with differential interference contrast (DIC) microscopy using a Nikon W1 spinning disk confocal system. (B) Supervised machine learning classification distinguishing elongated EVT cells from non-elongated cells based on morphology. (C) GFP signal used to generate nuclear masks for elongated EVT cells. (D) Tracking of elongated EVT nuclei over time to quantify directional migration. Scale bars = 200 μm.

B

A


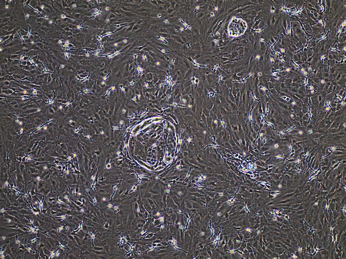

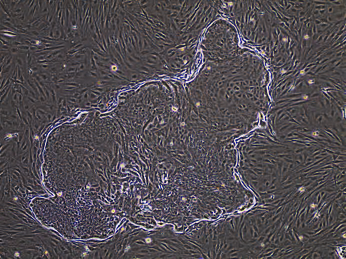

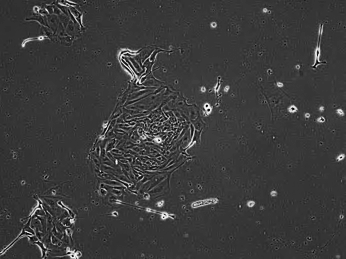

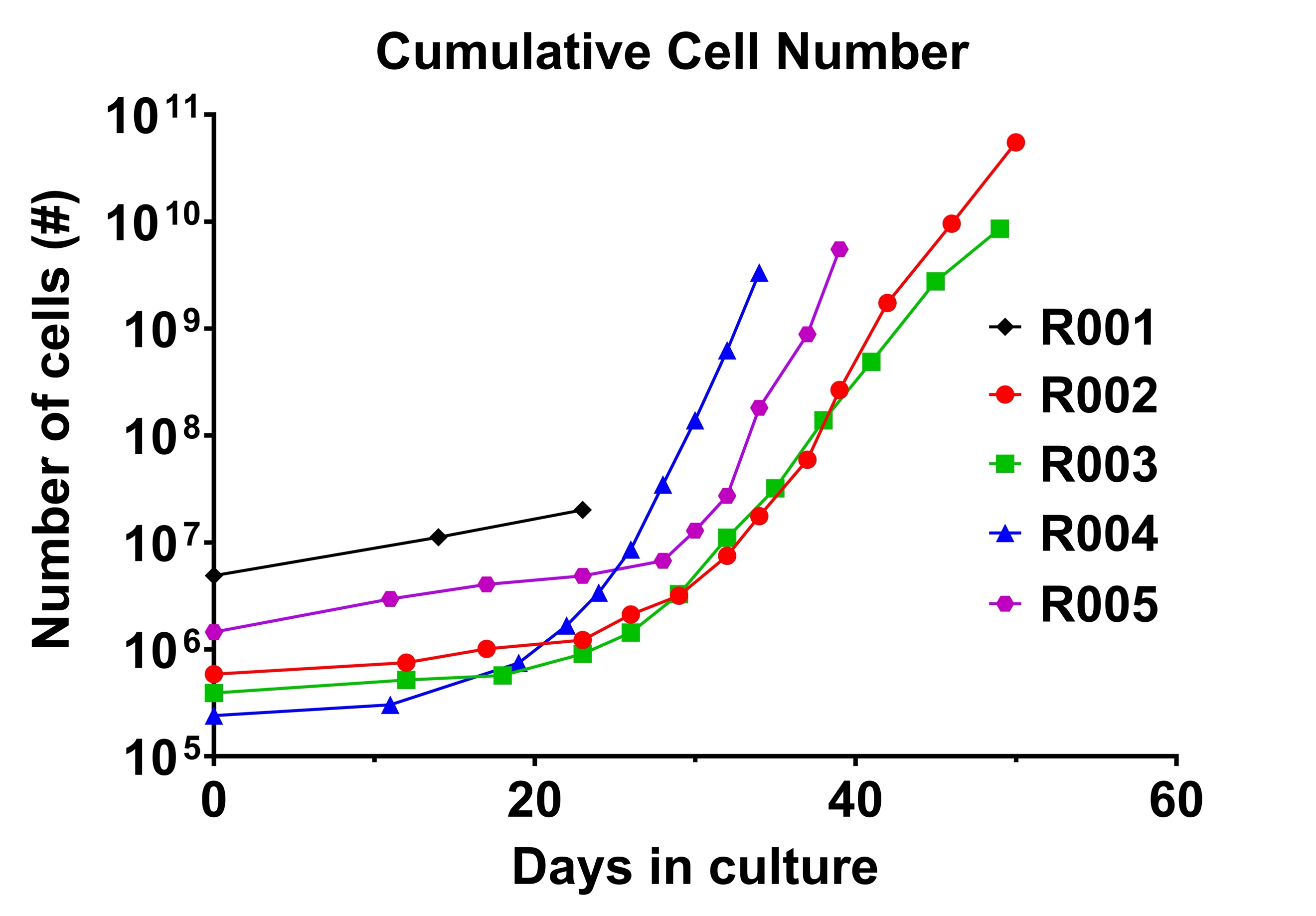


**Day 6**

**Day 19**

**Day 38**

**Passage 0**

**Passage 1**

**Passage 2**

**Supplementary Figure S3. Failure of trophoblast stem (TS) cell derivation from R001 products of conception (POC).** Trophoblast stem (TS) cell derivation was attempted from recurrent pregnancy loss (RPL) products of conception (POC) sample R001 but did not progress successfully. (A) Representative phase-contrast images showing cell morphology at passage 0 (P0), passage 1 (P1), and passage 2 (P2), with corresponding days in culture indicated. White arrows denote colony emergence. (B) Growth curve showing cumulative cell number over time, demonstrating failure to achieve exponential expansion. The dashed line indicates growth arrest at P2. Abbreviations: P, passage; POC, products of conception; TS, trophoblast stem. Scale bars = 250 μm.


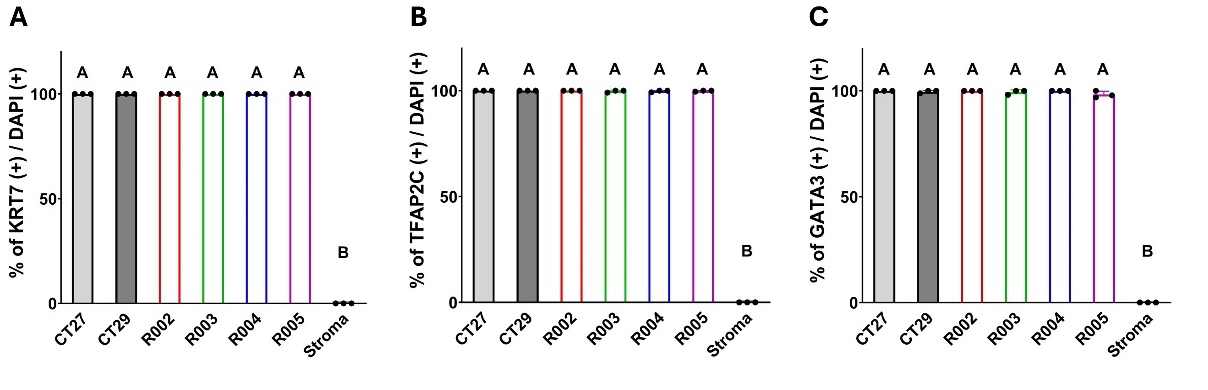


**Supplementary Figure S4. Expression of trophoblast markers in TS cells.** Quantification of trophoblast lineage marker expression in cytotrophoblast-derived TS cells (CT27, CT29) and RPL POC-derived TS cells (R002–R005). Percentage of cells positive for (A) keratin 7 (KRT7), (B) transcription factor AP-2 gamma (TFAP2C), and (C) GATA binding protein 3 (GATA3) relative to total nuclei (DAPI-positive; 4′,6-diamidino-2-phenylindole). Values represent mean ± standard deviation (SD; n = 3 biological replicates). Groups not sharing a common letter are significantly different (one-way analysis of variance [ANOVA], P < 0.05).


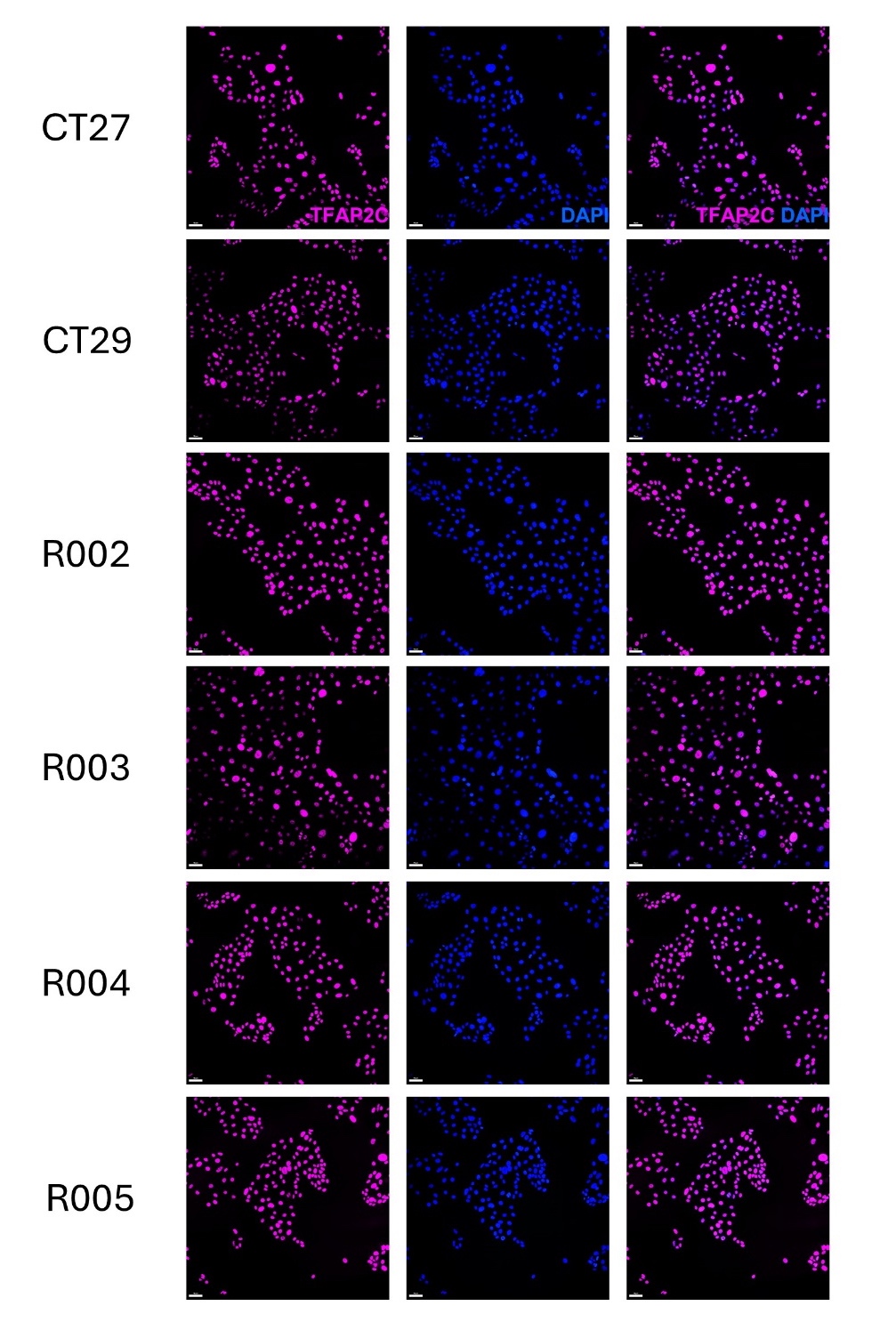


**Supplementary Figure S5. Representative TFAP2C immunofluorescence in TS cells.** Representative immunofluorescence images of transcription factor AP-2 gamma (TFAP2C; magenta) and nuclei stained with 4′,6-diamidino-2-phenylindole (DAPI; blue) in cytotrophoblast-derived TS cell lines (CT27, CT29) and RPL POC-derived TS cell lines (R002–R005). Scale bars = 50 μm.


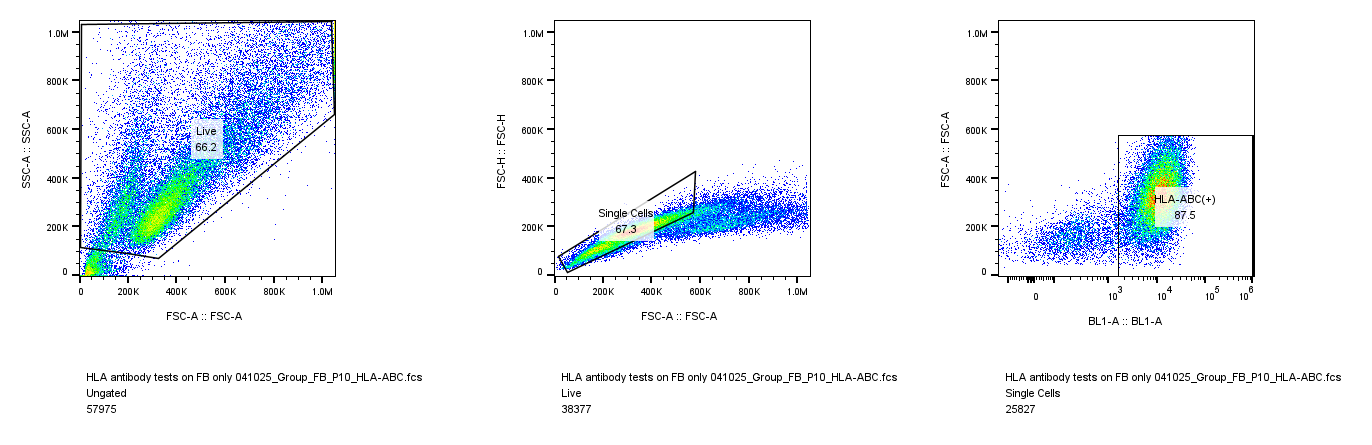


Live cells

Singlets

HLA-ABC(+) cells

**Supplementary Figure S6. Flow cytometry gating strategy for identification of HLA-ABC–positive cells.** Representative flow cytometry plots demonstrating the gating strategy used to identify human leukocyte antigen class I (HLA-ABC)–positive cells. Sequential gates include live cells, singlets, and HLA-ABC–positive populations. The defined HLA-ABC gate was applied consistently across all cytotrophoblast-derived and POC-derived TS cell samples for comparative analysis.


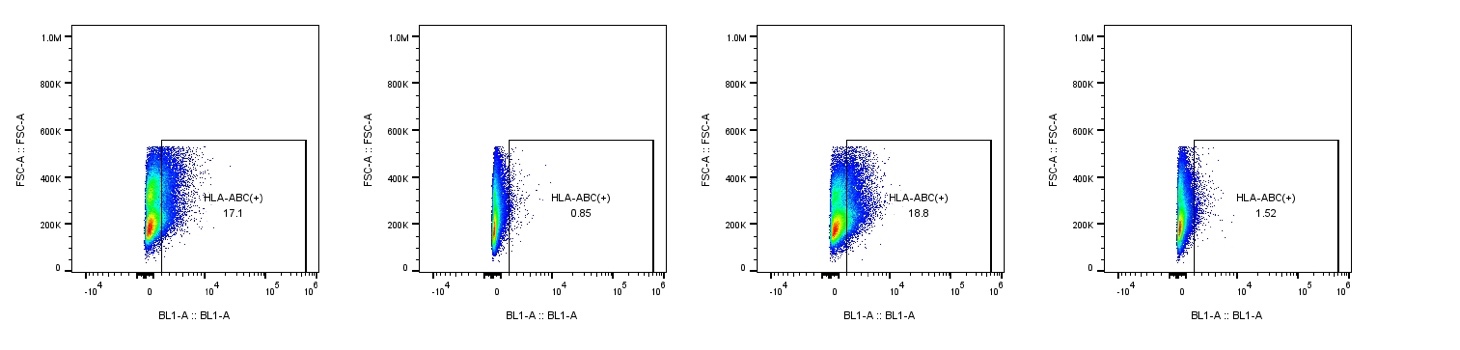

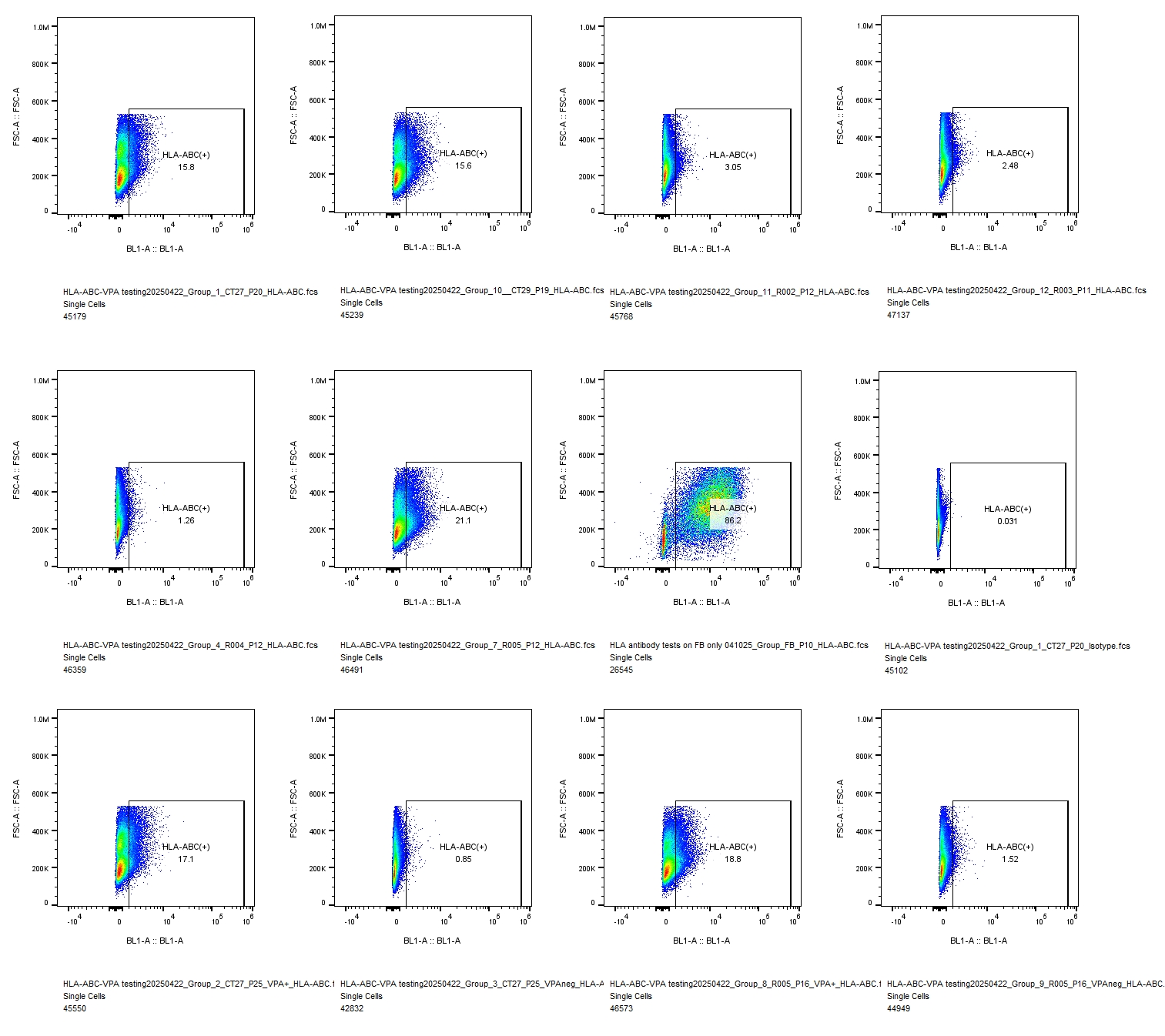

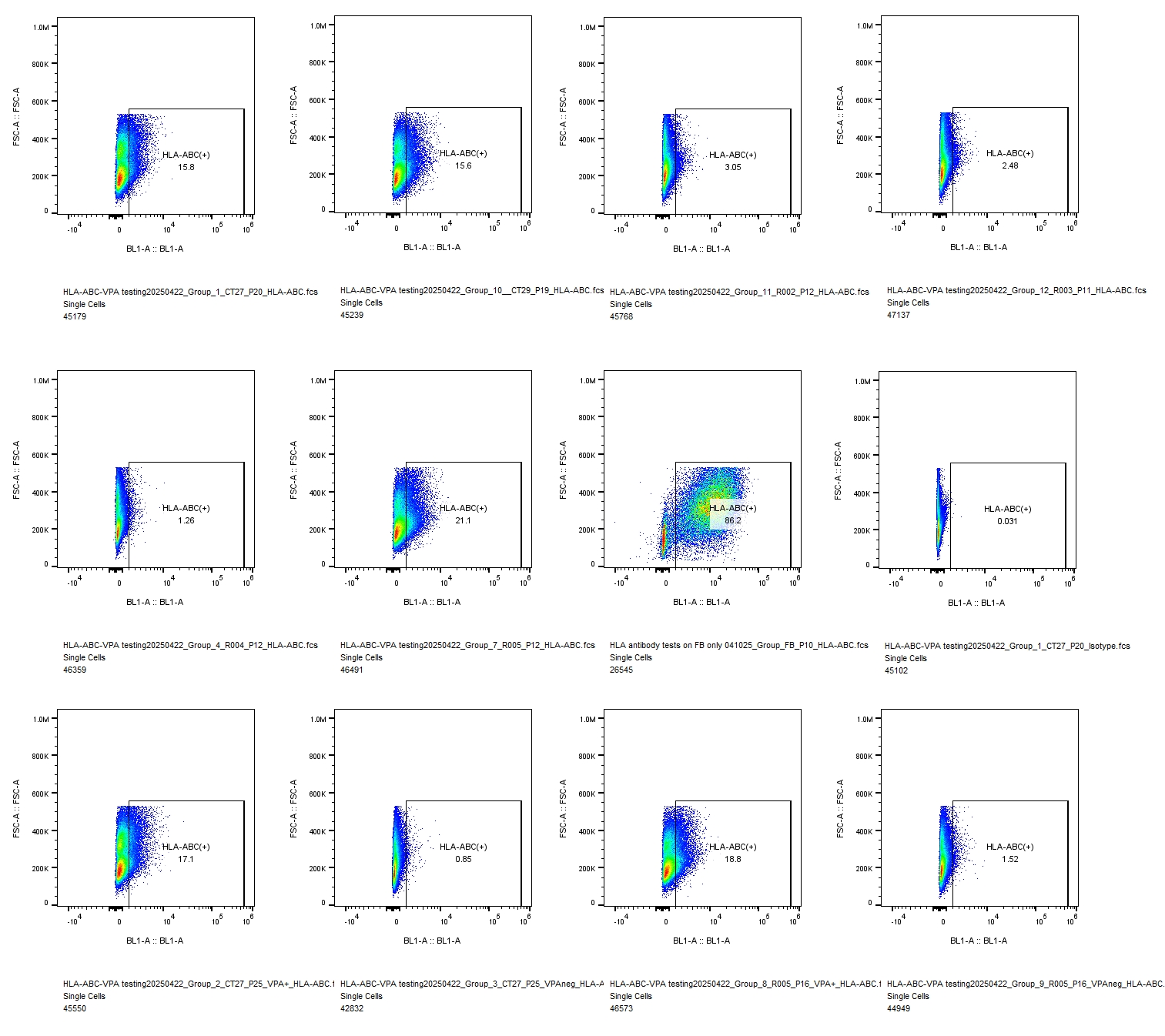


CT27 P20

CT29 P19

R002 P12

R003 P11

R004 P12

R005 P11

Stroma

Isotype Control

CT27 P25 VPA (+)

CT27 P25 VPA (-)

R005 P16 VPA (+)

R005 P16 VPA (-)

**Supplementary Figure S7. Quantification of HLA-ABC–positive TS cells.** Flow cytometric analysis of the percentage of HLA-ABC–positive cells in cytotrophoblast-derived TS cells (CT27, CT29) and RPL POC-derived TS cells (R002–R005). Data were analyzed using FlowJo software, and gating thresholds established in Supplementary Figure S6 were uniformly applied. Abbreviation: HLA-ABC, human leukocyte antigen class I.

** Supplementary Figure S8. Inverse relationship between spheroid size and number in STB differentiation.** Linear regression analysis assessing relationships between syncytiotrophoblast (STB) spheroid size and abundance in trophoblast stem (TS) cells. (A-C) Correlation between spheroid surface area and total spheroid count. (D-F) Correlation between spheroid volume and total spheroid count. Analyses were performed for (A, D) all samples combined, (B, E) cytotrophoblast-derived TS cells (CT27, CT29), and (C, F) RPL POC-derived TS cells (R002-R005). Coefficient of determination (R²) and P values are displayed in each panel. Negative correlations indicate that increased spheroid number is associated with reduced average spheroid size.

F

E

D

C

B

A

**Supplementary Table S1. Sample Metadata**

| **Sample ID** | **Genotype** | **Condition** | **Group** | **Sex** |
| --- | --- | --- | --- | --- |
| CT27-1 | CT27 | Control | Control | Female |
| CT27-2 | CT27 | Control | Control | Female |
| CT27-3 | CT27 | Control | Control | Female |
| CT29-1 | CT29 | Control | Control | Male |
| CT29-2 | CT29 | Control | Control | Male |
| CT29-3 | CT29 | Control | Control | Male |
| R002-1 | R002 | R002 | RPL | Male |
| R002-2 | R002 | R002 | RPL | Male |
| R002-3 | R002 | R002 | RPL | Male |
| R003-1 | R003 | R003 | RPL | Female |
| R003-2 | R003 | R003 | RPL | Female |
| R003-3 | R003 | R003 | RPL | Female |
| R004-1 | R004 | R004 | RPL | Female |
| R004-2 | R004 | R004 | RPL | Female |
| R004-3 | R004 | R004 | RPL | Female |
| R005-1 | R005 | R005 | RPL | Female |
| R005-2 | R005 | R005 | RPL | Female |
| R005-3 | R005 | R005 | RPL | Female |
