# Supplementary figures and images for: Functional heterogeneity in trophoblast stem cells derived from recurrent pregnancy loss products of conception
Source: Mol Hum Reprod. 2026 May 29;32(2):gaag033. doi: 10.1093/molehr/gaag033 (PMC13293087; doi:10.1093/molehr/gaag033)

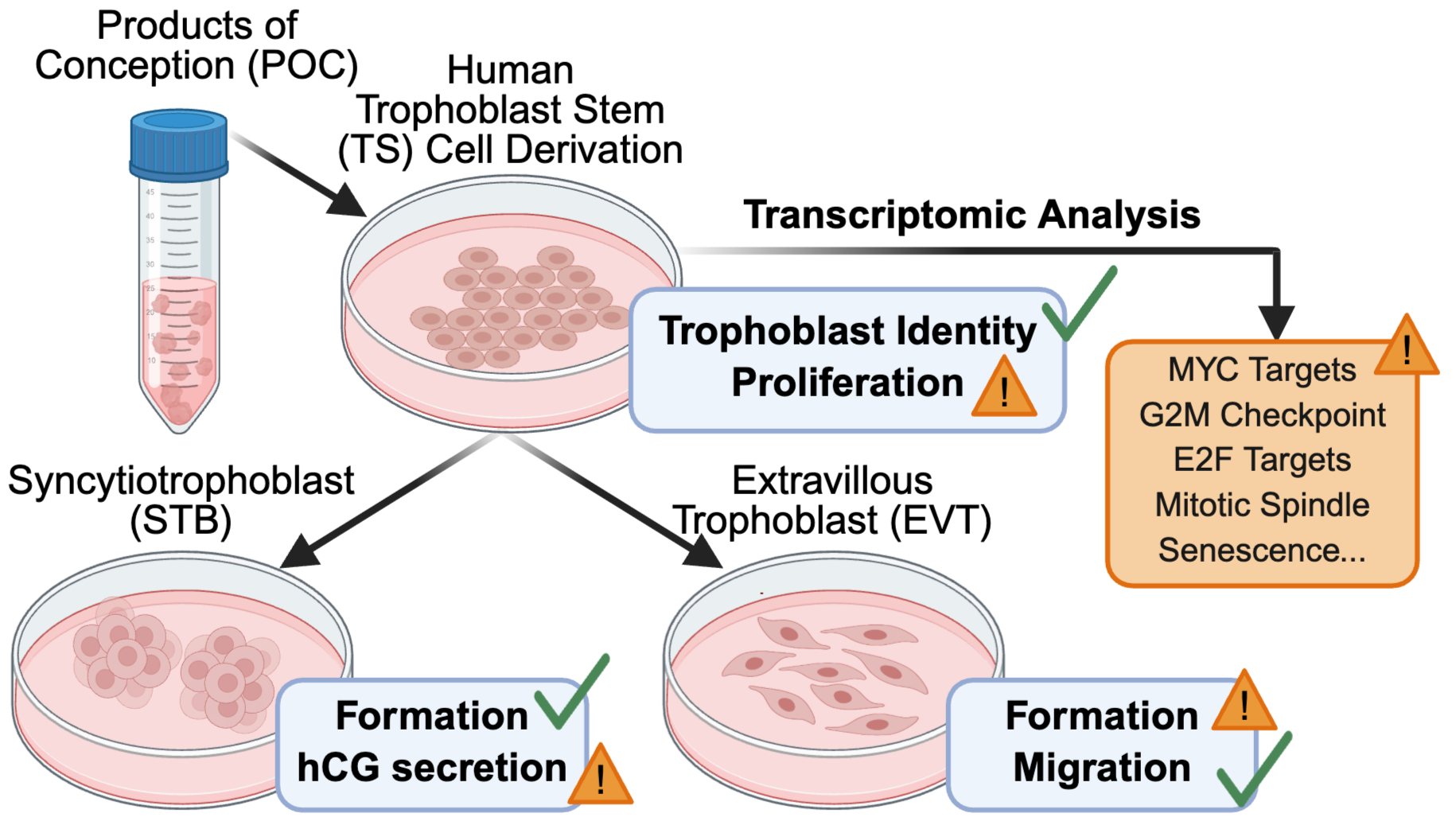

Supplement: gaag033_Supplementary_Data [file gaag033_supplementary_data.zip › RPL Graphical Abstract (1)-01.tif]
